# High-integrity forest carbon credits: Assessing equity and governance deficits in Colombia’s voluntary carbon market

**DOI:** 10.1007/s13280-025-02343-1

**Published:** 2026-02-25

**Authors:** Marcela Angel, Martin Camilo Pérez Lara, María Jimena Muzio, Angélica Mayolo-Obregón, Diego Casas, Juan José Mejía, John E. Fernández

**Affiliations:** 1 Environmental Research + Action ERA, Massachusetts Institute of Technology, 77 Massachusetts Avenue, Suite 5-418, Cambridge, MA 02139 USA; 2 Forest Team, WWF US, 1250 24th Street, N.W., Washington, DC 20037-1193 USA; 3 Environmental Solutions Initiative, Massachusetts Institute of Technology, 77 Massachusetts Avenue, Suite 55-106, Cambridge, MA 02139 USA; 4 Department of Architecture, Massachusetts Institute of Technology, 77 Massachusetts Avenue, Suite 5-418, Cambridge, MA 02139 USA

**Keywords:** Environmental governance, High-integrity credits, Indigenous Peoples, Afro-descendant and local communities (IPALC), Social equity, Voluntary carbon markets (VCM)

## Abstract

Voluntary carbon markets (VCMs) are crucial yet complex mechanisms for forest conservation, particularly in highly biodiverse regions. This study investigates the rapid expansion of carbon forestry projects (CFPs) across 26 million hectares in Colombia, with 92% overlapping indigenous peoples, Afro-descendant, and local communities (IPALC) territories. Using spatial and qualitative analyses, a novel IPALC participation ladder framework and Colombia’s first publicly available CFP geodatabase, we assess IPALC engagement dynamics, including market share, distribution, conflicts, and participation barriers. Findings show VCMs, despite significant conservation finance, often yield moderate IPALC participation, exacerbate governance deficits, and inequitable benefit sharing, compromising integrity, social legitimacy, and the potential for durable, community-driven conservation outcomes. Effective VCM climate action demands robust, community-driven governance, and inclusive policy reform. Lessons from Colombia offer critical insights for global VCM evolution toward more just, sustainable outcomes.

**Supplementary Information:**

The online version contains supplementary material available at 10.1007/s13280-025-02343-1.

## Introduction

Carbon forestry projects in voluntary markets (CFP) have emerged as a pivotal tool in the conservation and management of tropical forests. In the case of Colombia, due to their decentralized management characteristics, CFP have rapidly expanded to cover 26 million hectares of forests, equivalent to 22.7% of the country’s total continental area in less than 24 years. The Colombian market is primarily driven by reducing emissions from deforestation and forest degradation projects (REDD+), which represent the main source of carbon certificates and encompass 25.6 million hectares as compared to approximately 0,5 million hectares of afforestation, reforestation, and restoration projects (ARR). Indigenous peoples, Afro-descendant, and local communities (IPALC) in Colombia play a pivotal role in this market, particularly through the collective land management of 53.9% of the country’s forested areas, which includes the majority of REDD+ projects. These communities have historically been effective stewards of the land, through cultural and traditional natural resources conservation. However, their sustainable practices are increasingly threatened by the lack of viable economic alternatives and incentive structures, enabling extractive industries and illegal activities encroaching on IPALC’s territories, endangering their ancestral landscape management systems (FAO and FILAC 2021).

CFP offer an alternative source of income to the limited state transfers for IPALC, while opening new avenues for conservation and local economic development. To date, these projects have generated financial flows of USD 178.6 million and have the potential to generate an additional USD 404.6 million. CFP help bridge the gap between corporate environmental responsibility and establishing decentralized systems capable of reaching underserved territories. Yet, experiences of CFP in IPALC territories vary widely, requiring a systematic analysis to elucidate the factors shaping equitable and effective participation of IPALC in voluntary carbon markets (VCMs). While some projects have been reported to negatively impact community rights and governance structures, overshadowing social safeguards (Supplementary Information, Appendix B), others have successfully facilitated sustainable development and increased the agency of IPALC. Ultimately, ensuring long-lasting community-driven conservation outcomes from VCMs requires strong institutions that span scales, negotiate among stakeholders, regulate markets, enforce rights, provide transparency, deal with conflicts and that ensure deep community participation in both designing and overseeing these projects to avoid reinforcing social inequalities (Brown 2003; Miltenberger et al. 2021). Therefore, multiple international instances including the core carbon principles assessment framework and procedures (The Integrity Council for the Voluntary Carbon Market 2024), the VCM Joint policy statement and Principles (US State Department 2024), the UK government Principles for Voluntary Carbon and Nature Market Integrity (UK Department for Energy Security and Net Zero 2024), the Tropical Forest Credit Integrity Guide (Coordinator of the Indigenous Organizations of the Amazon Basin (COICA) et al., 2022), Article 6.4 of the Paris Agreement (UNFCCC 2015), among others, are calling for more transparency on use and management of revenues for benefit sharing and equitable agreements in VCM. Nonetheless, none of these have proposed specific recommendations on how to assess the relationships nor to refine VCM standards and processes to address inequalities.

## Synergies between the VCM, carbon tax, and collective land rights for IPALC

Colombia’s VCM has grown due to demand for carbon certificates from companies seeking to offset emissions (Pattberg and Stripple 2008, Mendieta and Grueso 2024) and the non-causation mechanism of the carbon tax. Established under Law 1819 of 2016, the carbon tax for 2024 is set at USD 5.96 per ton of CO2e, allowing companies to purchase carbon certificates from domestic mitigation projects in lieu of paying the tax. This mechanism has created a significant demand for carbon certificates in Colombia and is viewed as a key driver of the country’s VCM and its rapid market growth. However, institutional and regulatory challenges, including the suspension and reinstatement of the National Registry for the Reduction of Emissions and Removal of Greenhouse Gases (RENARE) and modifications introduced by Law 2277 of 2022—such as taxing coal and capping the non-causation mechanism at 50%—have impacted market growth. Moreover, the global VCM contracted significantly in 2023 (Proton 2024) due to substantial underperformance of the market associated with the quality of mitigation results (West et al. 2024), lack of transparency, and unfair distribution of benefits (Supplementary Information, Appendix B) and the resulting calls for improvements from civil society and human rights organizations, most notably resulting in Colombian Constitutional Court’s ruling for the Pira Paraná case (T-248 of 2024). These challenges underscore the need for stronger institutions and regulatory frameworks, enhanced transparency, and equitable access and benefit sharing as well as legitimacy in decision-making to realize potential development benefits and the integrity of the VCM (Brown 2003), particularly in a context where there is a significant overlap with IPALC territories.

Collective land titles and traditional land management practices have positioned IPALC as key actors in Colombia’s VCM. The country has a history of progressive policies recognizing IPALC’s civil rights, evolving from acknowledgment to special protection. Landmark legal instruments, including the 1991 Constitution, Law 70 of 1993 for Afro-descendant peoples (Congreso de la República de Colombia 1993), and various decrees such as 2001 of 1988 for indigenous peoples, have granted IPALC collective land ownership and jurisdictional autonomy (Benavides-Vanegas 2009). Additionally, Law 21 of 1991 ratified the indigenous and tribal peoples’ convention (C169), ensuring IPALC’s rights to consultation, land property rights and management, and cultural preservation (Corte Constitucional de Colombia 1997). This legal framework underscores the premise that land rights for IPALC are inextricably linked to the protection of human rights and the preservation of cultural identity, prioritizing the material sustenance and autonomy of communities over the exploitation of resources (Bárrios 2021; Bocanumenth Echeverri 2016). As of July 2024, IPALC collectively own 40.5 million hectares—35.4% of Colombia’s land—primarily in biodiverse regions like the Amazon and Pacific, where they manage over 24.6 million hectares of the Amazon (62.3% of the region) and 7.4 million hectares in the Pacific (58.5% of the region). Moreover, 53.9% of Colombia’s forests are located in IPALC’s territories.

Collective land tenure in Latin America is widely recognized as one of the most effective conservation mechanisms, often outperforming nationally protected areas (PAs), particularly in contexts of weak institutions characterized by low state presence, violence, limited capacity, poor provision of public goods, and remote geography. In Colombia, although effects vary across sub-regions, collective lands under weak institutions have reduced external pressures by limiting large-scale infrastructure and mining projects, lowering coca cultivation in some areas, and exhibiting consistently negative and significant deforestation coefficients (Bonilla-Mejía and Higuera-Mendieta 2019). Quasi-experimental studies confirm these effects: Afro-descendant peoples’ lands in Colombia, Brazil, Ecuador, and Suriname show 29–55% lower deforestation rates than comparable PAs, preventing the loss of about 60 000 hectares in the Colombian Pacific alone (Vélez et al. 2020; Sangat et al. 2025). In the Brazilian Amazon (2000–2021), indigenous territories and PAs together experienced only a small fraction of regional forest loss (Qin et al. 2023). In Peru, collective titling reduced deforestation by roughly three quarters and forest disturbance by two-thirds within 2 years, underscoring the importance of tenure security (Blackman et al. 2017). Similarly, titled Amazonian Indigenous Communities in Colombia, Bolivia, and Brazil achieved statistically significant reductions in carbon emissions—73%, 77%, and 90%, respectively—outperforming both strict protection and untitled indigenous territories (Blackman and Veit 2018). Where rights and resources are secured, community forestry can outperform strict protection, as shown in Guatemala’s Maya Biosphere Reserve, where community concessions maintained minimal deforestation while generating local income (Radachowsky et al. 2012). With historically lower deforestation rates and traditions of sustainable stewardship rooted in Indigenous governance systems (FAO and FILAC 2021; Semper 2006), IPALC’s collective land tenure and cultural resource management practices are central—but not sufficient on their own—for the success of CFP.

The current structure of carbon markets, which overlooks two key dimensions of equity, access and decision-making legitimacy, constrains their potential to deliver development benefits, and risks further excluding marginalized actors who may bear disproportionate costs (Brown 2003). Since its inception, Colombia’s VCM has been marked by innovative policies and continues to evolve rapidly, amid challenges of participation and IPALC governance in CFPs. To strengthen its market-based conservation instruments, Colombia is advancing in new regulatory and policy instruments such as the National Tradable Emission Quotas Program (PNCTE). Moreover, the current policy agenda aims to address critical issues related to social and environmental safeguards, human rights, governance and oversight, environmental integrity, baseline setting, additionality, carbon accounting, and the quality assurance of certification, validation, and verification processes for VCM. Similarly, the global VCM is at a pivotal moment. Regulatory frameworks and certification standards are evolving rapidly, while growing public pressure is reshaping expectations for transparency and community-centered approaches. By providing an assessment on the participation and governance barriers of IPALC’s in the VCM of Colombia, this paper provides a timely rationale for addressing these emerging challenges and proposes a series of recommendations to promote market integrity and social equity.

## Materials and methods

Our research, conducted from September 2023 to August 2024, employed a combination of spatial analysis and qualitative methods and pursued three research objectives: (1) provide a comprehensive overview of the market share and geographic distribution of CFP in Colombia relative to IPALC’s territories, strategic ecosystems, and reported conflicts in IPALC’s territories; (2) identify the main governance and institutional barriers faced by IPALC that hinder their effective participation in CFP, encompassing an assessment of the regulatory landscape, governance mechanisms, existing safeguards, progress made toward equitable benefit sharing, and project’s contribution toward the Sustainable Development Goals (SDGs); and (3) develop a framework to assess and strengthen equity dimensions in VCMs and apply it to the case of Colombia’s VCM. Consistent with these objectives, the study adopts a descriptive and exploratory approach, and does not seek to establish causal relationships regarding VCMs success or failure, but instead examines the conditions necessary to enable effective and equitable community-based conservation in the context of VCMs.

To address the first objective, we created the first publicly available comprehensive dataset and a geodatabase of CFP in Colombia (Available as a supplement to this article at Zenodo). The dataset draws on all five registries operating in Colombia, as well as datasets compiled by independent organizations including Berkley, ASOCARBONO, and WWF, and consolidates all identifiable CFP as of March 2024, resulting in 218 unique CFP project records. (Supplementary Information, Appendix A). We conducted spatial analysis on the full set, and calculated areas for the subset for which precise spatial information is available (170 project polygons). We developed several spatial indicators and a series of maps that provide a comprehensive overview of (i) the extent and regional distribution of CFP by type in Colombia; (ii) their overlap, by project type, with IPALC’s territories; (iii) overlaps with strategic ecosystems and protected areas; and (iv) the spatial distribution of projects with reported conflicts. We mapped projects with reported conflicts in IPALC territories to document the extent and geographic distribution of conflicts affecting IPALC, rather than to infer general impediments to VCM effectiveness.

To address the second objective, we conducted a qualitative analysis of the enabling and disabling factors affecting IPALC’s effective participation within Colombia’s VCM. This analysis included 20 semi-structured interviews with stakeholders across the VCM value chain, transcript coding, and thematic analysis across coding categories: general context, regulatory framework, benefit sharing mechanisms, governance and participation, safeguards compliance, and the potential contributions of CFP to the Sustainable Development Goals (SDGs). Interviewees were selected to ensure diverse representation across sectors, including 5 community representatives, 5 project developers, 2 validation and verification bodies, 1 standard setters, 2 registries, 2 policymakers, and 5 experts (academia, consulting, associations). With the exception of community representatives, who provided in-depth perspectives on specific projects, interviewees were selected for their extensive involvement across multiple CFP and VCM processes, enabling us to capture perspectives informed by broad, cross-project experience, rather than isolated project cases.

To address the third objective, we developed a theoretical framework to assess IPALC engagement in CFP and applied it to the case of Colombia’s VCM (See Supplementary Information for a detailed description of methods).

## Results

### Market share, extent, and regional distribution of carbon forestry projects in Colombia

**Fig. 1:**
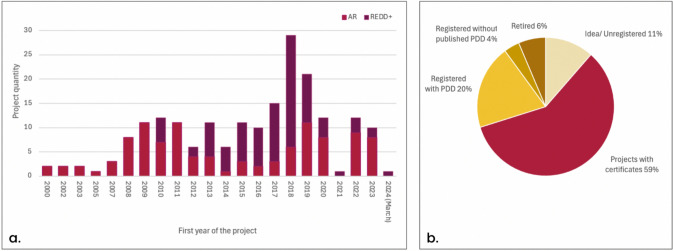
Projects by start year. **a** The number of projects started per year, based on the year reported as the beginning of activities in their PDD, broken down into REDD+ and ARR projects. **b** The percentage of projects by development status

CFP in Colombia began as early as 2000 with Afforestation and Reforestation (ARR) projects, followed by REDD+ projects starting in 2010. Figure 1 displays the number of REDD+ and ARR projects generated each year. Notably, the year 2018 stands out with the highest number of REDD+ project launches (14.7%), a surge that coincides with the implementation of the carbon tax’s non-causation mechanism, which is considered a key driver of Colombia’s VCM and its rapid growth.

Among the 218 projects, 128 have successfully generated carbon certificates, while 43 have formulated project design documents (PDD) but have not generated certificates. Consequently, as formulated projects undergo further verification and certification, an increase in supply is expected in the short term.

CFP represents a significant source of revenue for conservation. In total, 159 780 626 carbon certificates have been generated, with 48 926 302 already retired. Assuming an average price of USD 3.65 per certificate, it is estimated that these projects have generated USD 178.6 million and have the potential to generate an additional USD 404.6 million in the short term. Future market analysis should consider price changes due to the 68% reduction in transaction volumes for forestry and land-use projects, with REDD+ certificates being the most affected. Nonetheless, despite the reduction in transaction volume impacting both categories, ARR projects have experienced significant price increases (Donofrio et al. 2019).

Colombia’s VCM rapid market growth is illustrated by new private actors, with over 63 developers, seven carbon certification standards (BCR, CDM, CERCARBONO, COLCX, ICONTEC, Proclima, and VERRA), and 13 VVBs operating in Colombia over a period of 24 years and using a total of 29 methodologies, versions of methodologies, or combinations. This methodology heterogeneity combined with the lack of robust monitoring and oversight functions from public entities has created a complex scenario for monitoring compliance.

**Fig. 2:**
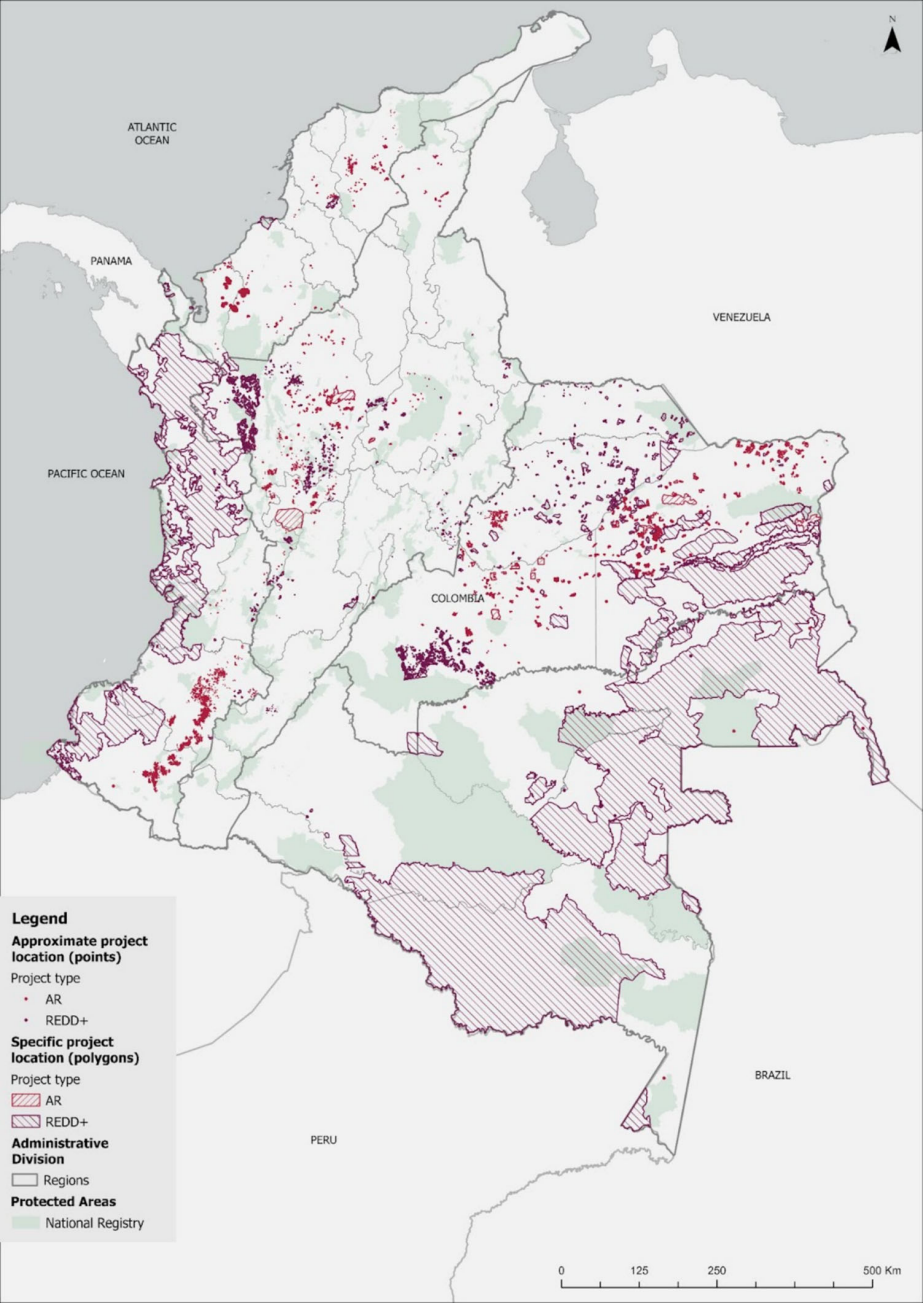
REDD+ and ARR projects and Nationally Protected Areas (PA) in Colombia. This map shows the areas covered by CFP categorized as REDD+ or ARR. Projects with defined boundaries are represented as polygons (170), while those with approximate locations are shown as points (48)

Most significantly, the VCM in Colombia has proven to be a fast-growing mechanism in terms of its coverage of large extensions of forested areas. Over a period of 24 years, CFP extended to cover 26 million hectares, equivalent to 22.7% of the country’s total continental area. Of the 218 projects identified, 108 are REDD+ and 110 are ARR projects. Nonetheless, REDD+ projects have been growing rapidly over the last 10 years and account for 98.4% of the total project area, with an overlap of 86 009 hectares between REDD+ and ARR projects. The Amazon and Pacific regions exhibit the highest concentration of projects, covering 44.5% and 34.6% of their area, respectively (Fig. 2).

### Extent and type of carbon forestry projects in IPALC’s territories

**Fig. 3:**
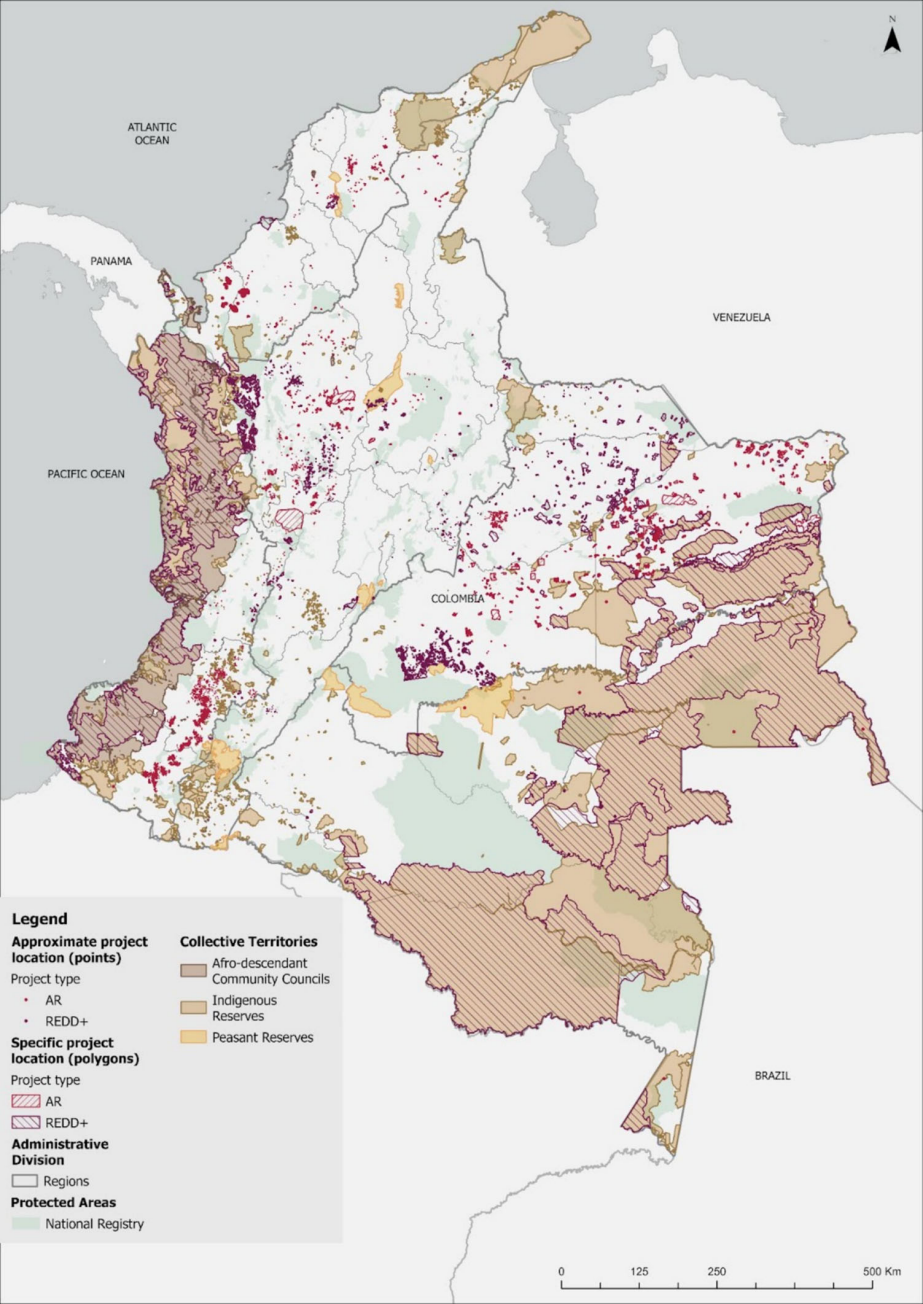
REDD+ and ARR projects and IPALC’s territories. This map shows areas covered by CFP, categorized as REDD+ or ARR, and their overlap with officially recognized Indigenous Reserves, Afro-descendant Community Council territories, and Peasant Reserves

The carbon forestry market in Colombia is largely a community-based market, with 92% of the area of CFP located in IPALC’s territories, equivalent to 24 million hectares. No ARR projects are reported within IPALC’s territories, with an overlap of less than 0.3% attributed to the adjacency of ARR projects to IPALC’s lands. Furthermore, REDD+ projects have been developed in almost 60% of all recognized IPALC’s collective territories. Specifically, REDD+ projects cover 60% of Indigenous Reserves, 71% of Afro-descendant Community Council territories, and 0.5% of Peasant Reserves. These are located within 71 Indigenous communities, 35 Afro-descendant community councils, and 7 Peasant Reserves. REDD+ projects also account for the largest share of carbon certificates, equivalent to 58,9% according to ASOCARBONO, the Colombian Association of Carbon Market Members, highlighting the country’s VCM reliance on certificates associated with IPALC’s lands and their long-term conservation vision and commitments (Fig. 3).

CFP through the REDD+ mechanism have become an unprecedented source of income for IPALC. In the Colombian Amazon and Pacific regions WWF and CCAP identified that annual financial revenue estimates range between 867 000 and 16 751 250 per project, typically lasting between 20 and 30 years (WWF Colombia and CCAP 2024). Considering that CFP are implemented in regions with both high and low deforestation rates, as well as diverse socio-cultural contexts and varying levels of territorial governance, there is potential for the VCM to expand rapidly into the remaining IPALC’s lands in a high-demand scenario.

### Strategic ecosystems overlap with carbon forestry projects

CFP cover 21 million hectares of forest, accounting for 37% of Colombia’s total forested area in 2022. Notably, projects also extend into other ecosystems and land uses, covering 4.77% of savannas, 0.06% of moorlands, and 2% of agroecosystems. Overall, PA encompasses 19.5 million hectares equivalent to 17.04% of the national continental territory, while the National Natural Parks System stands at 13.04%. Comparatively, despite having significantly different conservation mechanisms, it is notable that carbon forestry projects cover an area that exceeds the area of continental Nationally PA by 6,5 million hectares.

Our study detected an overlap of 1.6 million hectares between REDD+ projects and Nationally PA, raising concerns of double counting and overstated additionality. The additionality of REDD+ projects in these areas needs to be critically considered in light of increasing deforestation pressures. While legal protection prohibits forest clearing, it is often insufficient under weak institutions and high deforestation pressures. In these contexts, CFP could act as a complementary tool, whereas community-forest management provides local stewardship and social legitimacy, and REDD+ brings in financial resources to make conservation economically viable.

### Conflict analysis of carbon forestry projects in IPALC’s territories

**Fig. 4:**
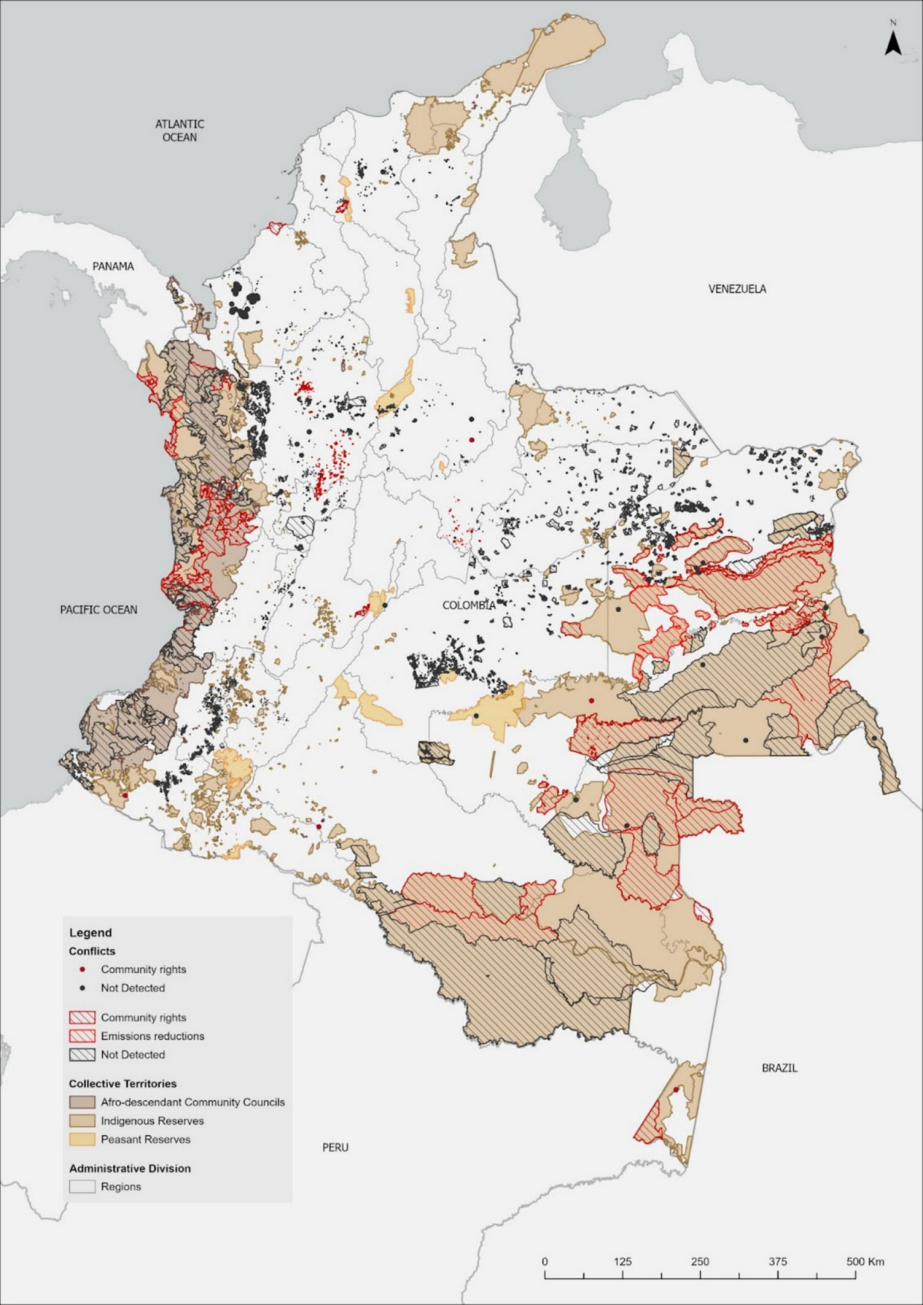
Carbon forestry projects in IPALC’s territories with reported conflicts. This map shows the overlap of CFP with reported conflicts with Indigenous Reserves, and the concentration of such projects in the Amazon and Orinoquía regions. The “not-detected” category represents projects without reported conflicts and should not be interpreted as an absence of conflict

Given the extensive involvement of IPALC in the VCM of Colombia, it is crucial to consider the active occurrence of conflicts in CFP with IPALC. In the absence of a public conflict-reporting mechanism or consolidated official data, media reports, particularly the investigative journalism of the Latin American Center for Investigative Journalism (CLIP), provide the most comprehensive information currently available on conflicts related to CFPs (Centro Latinoamericano de Investigación Periodística CLIP 2025). Nevertheless, despite CLIP’s systematic efforts, the inherent limitations of media-based reporting suggest that conflicts captured in this study are likely underreported (Fig. 4).

Through this media-based analysis, we identified 36 projects with community right conflicts and 1 with emissions reductions conflicts. Incorporating this information into our dataset and spatial analysis reveals that the majority of these conflicts are concentrated in the Amazon (19) and Pacific (8) regions. Of the identified conflicts, 21 are co-located with Indigenous Communities, eight with Afro-descendant Communities, two with peasant reserves, and five communities that could not be clearly identified.

Although only a relatively small proportion of projects report conflicts (37 out of 218), likely reflecting underreporting, these projects cover extensive areas, affecting 37.5% of the total CFPs area. As such, they have significant implications for IPALC rights. Across the sample, community rights conflicts most commonly arise from disputes related to land tenure, benefit-sharing arrangements, conflicts of interest among intermediaries, and legitimacy of decision-making processes.

### Enabling and disabling factors for IPALC participation: Challenges and best practices

This study identified six areas of interest associated with enabling and disabling factors affecting IPALC participation in the VCM, including general context, regulatory framework, benefit sharing mechanisms, governance and participation, safeguards compliance, and Sustainable Development Goals (SDGs). First, we outline interviewees’ perspectives on the challenges and disabling factors that shape the development of CFP (Supplementary Information, Appendix C). Second, we synthesize views on the enabling factors, best practices, and achievements related to each of the six areas of interest (Table 1).

**Table 1:** Summary of insights from interviewees on disabling and enabling factors for social impact in carbon markets, across key thematic areas

Category	Disabling factors and challenges	Enabling factors and best practices
General context	Armed conflict and illegal activities Accessibility barriers (complex procedures and language) Distrust in REDD+ projects Policy limitations (50% cap on non-causation mechanism) Lack of regulation for Law 70 Weak governance mechanisms within certain communities Lobbying against regulation Loss of traditional conservation practices	Collective land ownership over significant extensions of lands Cultural traditions for natural resources management Carbon tax and non-causation mechanism Higher education among community leaders Market consolidation as an ecosystem of actors
Regulatory framework	Limited institutional capacity for effective VCM functioning (operation of information systems; oversight and sanctioning; technical support for IPALC; and the implementation of prior consultation processes) Market failures (lack of transparency; information asymmetry; low-quality developers; misalignment with NDCs) Perverse incentives (baseline setting; selection of favorable standards and VVBs; profit-driven over fair benefit sharing; conflicts of interest) Gaps in regulation	Counterbalancing power asymmetries (Strengthened pre-project preparation phase; tools to verify overlaps; contract clauses on future sales; independent legal and advisory teams) Enhancing institutional capacities (Government-supported community training; unified information platforms) Investment models that reduce reliance on for-profit intermediaries Shift from flexible, market-oriented paradigm to human rights Adoption of investment models without for-profit actors
Benefit-sharing mechanisms	Lack of regulatory safeguards Transaction/verification opacity High financial barriers for IPALC and need for intermediaries Community governance weaknesses leading to territorial fracture (financial practices and education)	Transparency enhancing tools (full community ownership agreements; ombudsmen engagement; land-based distribution; benefit-sharing agreements based on final sale values; financial disclosure tools) Mechanisms to address entry barriers (Financial investment models to cover implementation costs while communities retain project ownership) Effective investment mechanisms (trust funds; accountability committees; alignment of project goals with community life plans)
Safeguards compliance	Lack of legal enforcement mechanisms and coordination in compliance control Inadequate social safeguards Misalignment between current interpretations with IPALC interests	Tracking tools for safeguard compliance Inclusion of safeguards in methodologies and contracts Social impact evaluations Government expansion of the National Safeguards System to the Agriculture and Forestry sector Ethics committees to investigate community complaints about projects Establishing communication channels with government agencies
Governance and participation	Power imbalances in negotiations (fraud and manipulation; information asymmetry; limited technical expertise and institutional support) Structural socio-economic barriers (structural violence; external pressures; gender inequality) Institutional instability Lack of consensus and control over the use of collective resources Insufficient resources to develop life plans and facilitate community assemblies	Strategic allyship (enhancing access to financial resources; strengthening collective decision-making through life plans development and public assemblies; technical and financial assistance) Accountability committees and trust funds with collectively agreed investment lines Community-driven agreements that support a just transition to conservation Empowered negotiation (ethics codes; education; third-party advisors)
Sustainable development goals (SDGs)	Armed conflict and illegal activities Structural socio-economic barriers Limited institutional capacity in IPALC territories Inadequate social safeguards Insufficient resources to develop life plan	Investments that support the transition to sustainable development models (green businesses, infrastructure, healthcare, food security, education, and governance) SDG-aligned project requirements (tracking tools, land titling incentives, and improved community governance and institutional coordination) Standards and registries developing impact measurement tools to track SDG compliance and social impact

### Engagement assessment framework: IPALC participation ladder for VCM

**Fig. 5:**
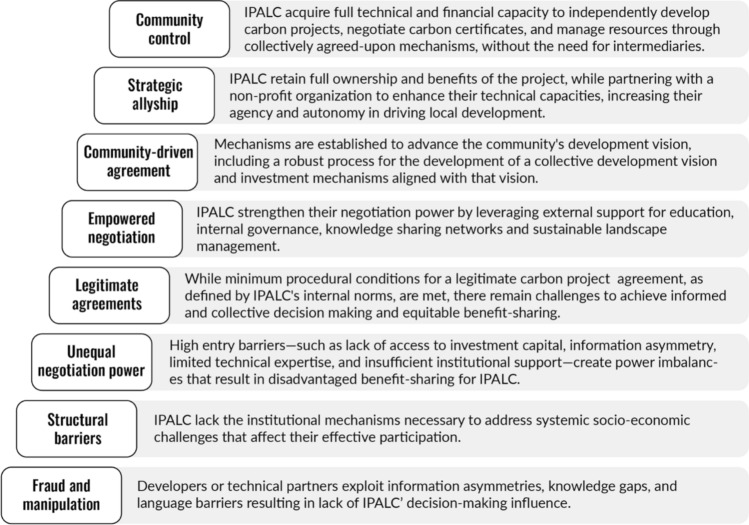
Participation ladder for VCMs. The ladder is a theoretical framework that includes a range of eight levels of engagement and associated conditions or participation mechanisms, ranging from non-participation at the lowest levels to full community control at the highest level

The conflict and qualitative analyses revealed a wide range of experiences and mixed outcomes regarding the participation of IPALC in Colombia’s VCM. These findings are consistent with the incentive structures of market-based conservation mechanisms, which constrain their potential to deliver development benefits and risks further excluding marginalized actors who may bear disproportionate costs. By favoring actors with greater landholdings, stronger social or political connections, or mechanisms that yield faster returns and administrative simplicity over participatory or inclusive processes, markets fail to ensure the inclusion of poorer, marginalized, or less powerful groups. In doing so, they not only neglect existing inequities but may also reinforce them (Corbera et al. 2007). To better assess these varying levels of engagement and provide a pathway to enhance IPALC participation, we propose a theoretical framework for IPALC engagement in VCMs. This framework draws on the citizen participation literature, particularly the Ladder of Citizen Participation, which conceptualizes levels of involvement in planning from non-participation to full citizen control (Arnstein, 1969). The framework is applied to the Colombian context to offer an overview of IPALC engagement in the country’s VCM (Fig. 5).

The participation ladder, the key element of this framework, is conceptualized as an eight-level continuum, based on conditions and participation mechanisms associated with varying levels of IPALC engagement in the VCM. The greatest challenges identified define the lower rungs of this ladder, where communities face fraud and manipulation or lack mechanisms or support to overcome structural barriers for their effective participation. As communities advance through subsequent stages of the ladder, their decision-making power within a project increases, gradually overcoming participation barriers, and developing self-organizing capacities to tap into the financial opportunities generated by CFP. At higher levels of the ladder, community norms and decision-making processes are enhanced as a result of their participation, ultimately leading to the highest stage, where communities fully control the design, implementation, and benefit sharing of carbon projects.

The participation ladder acts as a theoretical representation of the varying conditions and participation mechanisms identified in CFP in Colombia. As such, it provides a framework to assess IPALC engagement levels in other VCMs or in specific carbon projects, enabling the design of customized strategies and protocols to enhance community participation and control.

### IPALC participation ladder applied to the VCM of Colombia

This section summarizes the findings on governance and participation using the IPALC participation ladder for VCMs to map and rank the different experiences reported in the governance and participation excerpts. It provides an overview of the prevalent participation mechanisms and experiences in Colombia’s CFP, highlights variations in participation levels, and outlines a roadmap for enhancing IPALC engagement by showcasing mechanisms that support their advancement to higher rungs on the ladder.

1. Fraud and manipulation were discussed in 38 excerpts, referencing projects that have filed and in some cases won legal actions or that have been reported by the media for violations of community rights. In some of these cases, developers or technical actors target individual ethnic authorities, who have the authority to decide on a community’s participation in CFP, without using mechanisms that guarantee legitimate collective agreement or equitable benefit sharing.
2. Structural barriers for participation are widespread and were reported across 71 excerpts, describing internal governance instability—especially within indigenous communities; lack of participation mechanisms such as assemblies for collective decision-making or committees for overseeing resource management; insufficient resources to convene members across vast Indigenous territories; limited trust and legitimacy of ethnic authorities; lack of a cohesive development vision or incomplete life plans; absence of governance norms to manage CFP revenues and to prioritize investments based on collective priorities; external threats and pressures that incentivise extractive activities and perpetuate structural violence; and in some indigenous communities, limited participation of women.
3. Unequal negotiation power has been reported in 18 excerpts referring to projects across all regions and among IPALC. Developers, who hold the total investment capital to launch a project, wield disproportionate influence over project design, benefit-sharing negotiations, and carbon certificates commercialization. This imbalance is exacerbated by information asymmetries, with developers or technical actors possessing insider technical and financial knowledge from their involvement in multiple deals. Moreover, the limited government support for communities often creates dependency on information and technical assistance provided by developers, which may be biased. The public ministry responsible for safeguarding IPALC’s rights also lacks sufficient technical expertise to effectively weigh-in in certain decisions.
4. Legitimate agreements are widely discussed across 69 excerpts and describe project agreements that meet the minimum procedural standards for legitimacy, ensuring that decision-making bodies adhere to the internal norms required for prior and informed consent. This includes ensuring that any assembly or consultative body with decision-making power over CFP has the minimum quorum required by the internal norms; overcoming common barriers for participation such as the geographical distance between population centers, language barriers by translating materials into local languages and culturally appropriate formats, and the provision of resources to support the minimum number of required meetings. Nonetheless, legitimate agreements alone do not suffice to guarantee informed participation, as they fulfill procedural requirements but may not provide the technical knowledge needed for IPALC to make informed decisions.
5. Empowered negotiation is reported across 52 excerpts that describe projects where developers, government agencies, VVBs, and sectoral associations collaborate to provide comprehensive technical assistance to IPALC. In these cases, IPALC leverage project resources to strengthen governance structures, adjust or develop life plans, and create internal norms tailored to the new financial opportunities generated by CFP. Technical assistance may come from third-party experts selected by the community, educational materials and training offered by the government, or nudges and inquiries from VVBs aimed at raising awareness about benefit sharing. Some projects have implemented codes of conduct and ethics developed by sector associations to regulate the relationships between developers and IPALC.
6. Community-driven agreements are discussed across 20 excerpts and encompass projects with profit-driven developers that have been awarded or recognized for their positive impact on IPALC. These projects are characterized by broad community consensus around a development vision, often created through a robust life plan development process, and by focusing the investment of carbon project’s resources on implementing the life plan goals. These projects have made significant investments to strengthen community governance, implemented financial instruments such as trust funds for transparency, established community-led accountability committees to oversee investments, and conducted assemblies with oversight from the public ministry.
7. Strategic allyship is described in 6 excerpts that illustrate how international cooperation acts as a non-profit partner for Indigenous and Afro-descendant communities, helping them overcome entry barriers by providing necessary resources and conducting a comprehensive community preparation process to strengthen internal governance, and local technical and financial capacities. Through these projects, communities receive grants via a revolving fund to cover initial capital investment while retaining full project ownership. The preparation process includes investments in self-organizing capacities, participatory planning mechanisms, and training programs that enhance IPALC’ market knowledge, financial literacy, and sustainable landscape management, specifically focused on supporting the transition from deforestation and illegal activities to sustainable development models, promoting green businesses, ecotourism, and agroforestry.
8. Community control is the highest level of participation in the theoretical framework, where communities have full ownership and autonomy over all aspects of a CFP—design, implementation, and commercialization—without intermediaries. Although it was described across 13 excerpts, it was presented as a goal to be achieved and would require IPALC to possess the necessary investment capital, market knowledge, and technical expertise, giving them full control and decision-making power over every aspect of the project. Under current market conditions, no CFP in Colombia has yet achieved this level of IPALC participation.

The use of the IPALC participation ladder for VCMs to analyze the case of Colombia suggests that (1) there is a wide range of experiences and the current market dynamics, incentive structures, and policy gaps tend to favor projects that remain in the mid-levels of the participation ladder. With structural barriers (71 excerpts) and legitimate collective agreements (69 excerpts) cited as the most common experiences, followed by empowered negotiation (52 excerpts) evidence suggests that the challenge is not isolated but systemic, as a number of initiatives have not addressed social structural barriers. However, there are relevant efforts from various actors who are actively working to overcome these challenges through diverse participation mechanisms; (2) despite ongoing efforts that mandate safeguards’ compliance, those have not been sufficient to prevent fraud and manipulation (38 excerpts), which remain as significant concerns; (3) although there are less accounts of experiences in higher engagement levels, a couple of cases of community-driven agreement (20 excerpts) and strategic allyship (6 excerpts) emerge as viable alternatives to profit-driven model. These are projects that have been implemented successfully and are positioned toward the higher levels of the ladder providing examples of best practices and mechanisms for achieving equitable benefit sharing and more effective participation; (4) Community control, the highest level of participation on the ladder, remains largely a theoretical concept and may not necessarily be the optimal level of control for IPALC at the individual level, as individual communities may fail to capture the potential benefits of economies of scale and collective knowledge. Nonetheless, community associations are particularly well positioned to collect, analyze, and share the type of experiential and collective knowledge that could enhance systemic community control; (5) regardless of the level in the ladder, interviewees emphasized that IPALC participation is closely linked to the governance capacities of the communities as well as material conditions that determine their ability to manage projects and access their benefits.

Consistent with the evidence, participation in CFP depends not only on material conditions such as land endowment, land quality, and production dynamics, but also on broader social development indicators, including secure property rights and tenure, strong local institutions, and existing forest management capacities (Corbera et al. 2007). Communities with stronger assets in these areas are better positioned along two fundamental dimensions of equity. The first is access: who can use land and forest resources for livelihoods, and who can enter carbon markets or benefit from carbon credits through secure property rights, technical capacity, access to information, and lower transaction costs. The second is decision-making legitimacy: who participates in governance, how decisions are made, whose voices are heard, and what institutions exist to support inclusion and fairness (Brown 2003). Strength across these dimensions increases the likelihood that communities can self-organize to capture benefits from carbon projects and negotiate community-driven agreements, aligning CFP objectives with local development and conservation priorities.

Variation in levels of community control and participation is further associated with differences in social capital, leadership style, and internal organization. Research on community forestry governance highlights the importance of intangible factors such as members’ emotional attachment and loyalty to local institutions, as well as a shared collective identity. Participatory leaders who embody community values and maintain close ties with members, tend to strengthen engagement in forest management and conservation (Sinha and Suar 2005). Accordingly, communities’ ability to ascend the participation ladder depends not only on project design, but on how effectively CFP are leveraged to strengthen inclusive leadership as well as internal governance mechanisms and norms, and on the extent to which equity is embedded in decision-making processes that increase communities’ ability to influence project rules, benefit-sharing agreements, and long-term land-use implications. These conditions are essential for ensuring durable community-driven conservation outcomes as new income streams become available to IPALC.

Taken together, these findings suggest that focusing on participation barriers and governance challenges is well justified, as these dimensions shape the distribution of opportunities, information, and decision-making power within VCM arrangements. However, available evidence does not support the argument that such challenges necessarily lead to the failure of VCM initiatives. Rather than signaling inevitable project underperformance, our findings indicate that governance and participation function as enabling conditions that mediate how market incentives translate into equitable outcomes, legitimacy, and long-term effectiveness of the community-driven conservation efforts on which critical tropical forested regions, such as the Amazon or the Pacific, have historically relied. Weak or uneven governance capacities can constrain communities’ ability to access, negotiate, and benefit from carbon finance, whereas robust participatory structures can enhance transparency, strengthen accountability, and support more durable and inclusive community-driven conservation models.

## Discussion

### Multi-faceted conservation

The literature increasingly emphasizes that no single policy or incentive is a “silver bullet”. PA, indigenous land rights, and REDD+ economic incentives should be viewed as *complementary*, not mutually exclusive, strategies (Börner et al. 2020). Each addresses different facets of the deforestation problem and their effectiveness often hinges on being used together.

IPALC collective tenure areas are key to forest conservation under weak institutional contexts, exhibiting far less deforestation than unprotected areas (see section Synergies between Synergies between the VCM, Colombia’s Carbon Tax and Collective Land Rights for IPALC). This success is often attributed to strong traditional stewardship, local governance, and the fact that granting land tenure to communities removes the threat of external land grabs. One might argue that if an indigenous community has historically kept deforestation, a carbon project that pays them to continue may lack *additionality*, since the expected deforestation without the project could be so low that the project’s claimed emissions reductions are inflated. However, many community forests are facing new threats (e.g., advancing agricultural frontiers, mining, or illegal logging as markets penetrate remote areas, lack of economic alternatives and lack of economic alternatives, or insufficient capacity for effective forest management). Additionally, communities often lack funding for effective surveillance or sustainable development. In these contexts, REDD+ projects can provide critical financial incentives, livelihood improvements, and enforcement support that help communities resist outside pressures and maintain their forests (Alejo et al. 2022).

In other words, carbon finance can reinforce and sustain Indigenous stewardship that might otherwise be undermined by competing economic pressures. Our interviews indicate that CFP frequently fund development goals such as education, healthcare, sustainable enterprises, and training in monitoring and governance, which enhance project additionality by making forest conservation more valuable to communities than deforestation-driven alternatives (Pauly et al. 2022). Consistent with our participation ladder findings, projects in collective territories are more likely to be genuinely additional when they target frontier areas facing emergent pressures and when revenues tangibly strengthen communal governance, monitoring systems, and livelihood options that shift the business-as-usual trajectory.

Similarly, REDD+ could help finance conservation in PA but proving additionality is more challenging, given legal mandates to maintain forest cover. Many PA were historically placed in “bullet-proof” low-pressure locations (e.g., remote or less arable lands) to avoid conflict, which limited their immediate impact but also avoided the hardest challenges (Börner et al. 2020). PA proven capacities to reduce deforestation as compared to non-protected areas, but failure to reduce deforestation rates as effectively as collective lands under weak institutions indicates that mandates alone are limited as conservation mechanisms. Recent causal evaluations show that voluntary REDD+ projects can reduce deforestation when they operate in high-pressure contexts and when baselines, leakage, and permanence are conservatively addressed (West et al. 2023). Acknowledging the limitations of PA, Colombia’s CFP should prioritize project development where threat is demonstrably high rather than in low-risk protected areas, either in collective territories or PA. Where additionality is credible and governance aligns with protected area authorities, it could bridge funding gaps for enforcement, community co-management, and restoration.

### Multi-scale and impact-oriented intermediaries

Our dataset reveals a proliferation of intermediaries including developers, aggregators, multiple standards, and VVBs, alongside conflicts of interest and information asymmetries that correlate with mid-level participation. We also document 37 conflicts (37.5% of projects), largely linked to community rights violations in indigenous territories. This pattern mirrors peer-reviewed findings that intermediaries shape REDD+ outcomes through rule-making and brokerage, with benefits contingent on capacity, accountability, and institutional fit (Kim et al. 2016; Corbera and Schroeder 2011). Studies of the “social life” of forest carbon similarly show how contracting and intermediation determine value capture and accountability to rightsholders (Mahanty et al. 2012). Empirical work underscores that secure tenure is a prerequisite for equitable outcomes (Sunderlin et al. 2018) and that intermediary performance depends on state capacity and clear mandates (Kim et al. 2016). Likewise, comparative studies highlight that benefit-sharing design is central to legitimacy and cooperation, as perceived unfairness erodes project support (Luttrell et al. 2013). Concentration of CFP in the mid-ladder pattern (procedurally “legitimate” agreements without consistent empowerment), reflects these governance dynamics and illustrates why a community-centered but intermediary-intensive market continues to produce uneven equity outcomes. Taken together, our conflict sample and mid-ladder participation levels reinforce these findings and point to the need for stronger public oversight of contracting, standardized minimum terms for benefit sharing, and accessible grievance mechanisms to reduce power asymmetries between IPALC and private developers.

The literature indicates that projects led or validated by non-profit organizations align more closely with actual emission reductions than those involving for-profit entities. Non-profit-led projects report average offset-credited alignment (OAR) rates more than twice as high as their for-profit counterparts, with the effect even stronger when both the project proponent and validator are non-profits (Probst et al. 2025). These findings highlight the multilayered influence of organizational type and collaborative structure on the integrity of credited carbon emissions. Complementing this, an analysis of equity and development in the carbon economy in Mexico concludes that achieving sustainability and equity goals requires institutions that operate across scales (local, regional, national, international) to negotiate among stakeholders, regulate markets, ensure participation, enforce rights, promote transparency, and address conflicts (Brown 2003). Absent such institutional frameworks, markets alone are often insufficient to guarantee equity.

Our analysis of projects in the higher participation levels of the ladder, where non-profit developers effectively reduce entry barriers by providing necessary resources and conducting a comprehensive community preparation process to strengthen internal governance, local technical, and financial capacities, underscores the development benefits of multilayered, impact-oriented organizations. Addressing equity concerns in Colombia would benefit from formalizing intermediary roles and establishing public guidance on minimum benefit-sharing terms and disclosure, while strengthening IPALC leadership to move projects upward on the participation ladder. International markets can reinforce these shifts by conditioning demand on independent evidence of additionality and equity, and by rewarding tenure-secure, high-threat projects that deliver co-governed benefits to rightsholders (Nolte et al. 2013; Blackman et al. 2017; West et al. 2023). Importantly, our interviews indicate that additionality in these contexts depends on whether carbon finance measurably strengthens governance, monitoring, and livelihood alternatives under emerging pressures, rather than simply compensating historically low deforestation.

## Conclusions

Drawing from the qualitative research process, spatial analysis, and the newly proposed theoretical framework—the IPALC Participation Ladder—this study offers the most detailed account to date of how Colombia’s VCM is operating in practice in relation to IPALC.

The study demonstrates that Colombia’s VCM has undergone rapid territorial expansion, with CFPs now covering 26 million hectares, equivalent to 22.7% of the country’s continental area. The vast majority of these projects are REDD+ initiatives developed in IPALC territories, which collectively represent 92% of the total area covered by CFP. This dynamic has positioned Colombia as a global reference for decentralized climate finance, where collective land tenure and IPALC governance play a central role in climate mitigation efforts.

The rapid growth of CFPs and overlap with IPALC territories, however, has yielded mixed outcomes and often only moderate levels of IPALC participation. This mid-level engagement often involves consultation and the minimum conditions for legitimate agreements rather than genuine decision-making legitimacy and power for IPALC. While some projects have contributed to sustainable development and empowered communities to implement their development visions (IPALC Participation Ladder: strategic allyship, community-driven agreements, and empowered negotiation), others have created or exacerbated governance challenges, weakened institutions, and resulted in inequitable benefit sharing (IPALC Participation Ladder: unequal negotiation power, structural barriers, fraud, and manipulation). Crucially, this study demonstrates that the effectiveness and equity of carbon markets hinge not only on technical design, legal frameworks, or quantitative participation mechanics, but also on the quality of participation, governance capacities and alignment with community-led development visions. Material assets, secure rights, and strong local institutions shape who can access markets and how benefits are shared, while leadership and social capital determine decision-making legitimacy. Carbon projects advance conservation and equity only when they strengthen these community foundations and embed fairness in governance.

This study’s findings, synthesized across seven thematic areas, highlight several interrelated discussion points: (1) extent and scale; (2) reaching of historically underserved regions; (3) the foundational role of collective territories; (4) power asymmetries, intermediaries, and the regulatory void; (5) governance as a determinant of project success; (6) transient nature of developers; (7) alternative development models.

1. *Extent and scale* Colombia’s VCM now covers more land than the national system of PA, demonstrating the potential of CFP to rapidly mobilize conservation finance. However, this expansion raises concerns about market structure and equity considerations, and the appropriate scale of market-based solutions within the portfolio of climate and conservation finance mechanisms. The VCM’s decentralized and rapid growth has resulted in broad reach but uneven outcomes, strongly influenced by profit-driven intermediaries and regulatory gaps, local governance, and material conditions of IPALC.
2. *Reach of historically underserved regions* A key contribution of Colombia's VCM is its capacity to direct investment to historically underinvested regions under weak institutions characterized by low state presence, violence, limited capacity, poor provision of public goods, and remote geography. In areas such as the Amazon and Pacific, CFPs have enabled access to new financial flows and opened possibilities to advance critical sustainable development goals related to education, infrastructure, and basic services. However, these benefits are unevenly distributed and often hard to materialize, and in the absence of state oversight, conflict resolution, and accountability mechanisms, communities are often left to negotiate critical aspects of their development visions with actors holding disproportionate power.
3. *Foundational role of collective territories* Colombia’s VCM relies heavily on IPALC territories: 92% of CFP areas are located within them, and over half of indigenous and Afro-descendant collective lands host REDD+ projects. Consequently, the market’s success depends on IPALC stewardship and IPALC finance streams are increasingly connected to CFP, necessitating a shift in perspective and a regulatory framework that centers equity dimensions of access and decision-making legitimacy, removing entry barriers and creating incentives for IPALC positionality as architects and owners, not just participants, in climate mitigation and development strategies within their territories.
4. *Power asymmetries, intermediaries, and a regulatory void* This study reveals significant power imbalances between developers, technical actors, and IPALC, due rule-making and brokerage capacities of developers and OVVs, exacerbated by limited state capacity, clear conflict of interest regulations and enforcement; lack of accountability to rightholders; uneven technical and finance capacity to materialize value capture; and the material conditions and structural inequalities faced by IPLAC. To address these gaps, we propose three critical regulatory institutions: a certifying agency for market actors, an environmental regulatory commission for standards and baselines, and a superintendence for enforcement and accountability.
5. *Transient nature of developers* Developers were key to the VCM’s initial expansion. However, as the market matures and IPALC, supported by multi-scale impact-oriented intermediaries, strengthen their governance and technical skills, a shift should occur: developers’ roles should diminish, and benefit sharing for IPALC should increase.
6. *Governance and material factors as a determinant of project success* Applying the IPALC participation ladder framework, the study finds that strong community governance is closely linked to effective participation. Communities with robust life plans, assemblies, oversight committees, and financial literacy tend to achieve higher participation levels that result in better outcomes from their engagement in VCM. Conversely, weak governance increases the likelihood of benefits being misaligned with collective priorities or captured by external actors. Thus, reinforcing IPALC governance must be a primary investment in CFPs, material conditions such as land endowment, land quality, and production dynamics (Corbera et al. 2007), but also on broader social development indicators including income, secure property rights, and existing forest management capacity.
7. *Alternative development models* The study challenges the notion that economic development in forested areas must rely on extractive industries coupled with environmental degradation, and argues that multifaceted conservation tools along carbon finance can reinforce and sustain indigenous stewardship that might otherwise be undermined by competing economic pressures. This fosters local economies centered on green businesses, agroforestry, and ecotourism. However, success hinges on aligning investments with local priorities and ensuring equitable resource control.

Colombia’s VCM presents both promise and caution. It has generated income, spurred innovation, and showcased the potential of decentralized climate and conservation finance. Yet, it also exposes deep structural challenges, weak governance and regulation gaps, power imbalances and conflicts of interest, and limited participation. To realize its potential, the market must evolve through investments in IPALC governance, regulatory reform, enhanced transparency, and the development of community-centered models. Only then can carbon markets become genuine instruments for equitable climate action and sustainable development.

## Supplementary Information

Below is the link to the electronic supplementary material.

Supplementary file1 (PDF 726 KB)

## Data Availability

The geodatabase of CFP and GIS layers generated and analyzed in this study is available at Zenodo public data repository. Country and regional boundaries are freely available at https://www.dane.gov.co/files/geoportal-provisional/index.html (Departamento Administrativo Nacional de Estadística (DANE) 2018); IPALC territories data is openly available for Afro-descendant territories at bit.ly/ANT-ConsejoComunitarioTitulado (Agencia Nacional de Tierras (ANT) 2024a), Indigenous Reserves at https://bit.ly/ANT-ResguardoIndigenaFormalizado (Agencia Nacional de Tierras (ANT) 2024b), and peasant reserves at https://bit.ly/ANT-ZRCampesinaConstituida (Agencia Nacional de Tierras (ANT) 2024c); ecosystem data is available from bit.ly/3XEDKPW (Instituto de Hidrología, Meteorología y Estudios Ambientales (IDEAM) 2017); forest data is available from IDEAM upon request (Instituto de Hidrología, Meteorología y Estudios Ambientales (IDEAM) 2022); protected areas and national parks data are freely available at https://runap.parquesnacionales.gov.co/cifras (Registro Único Nacional de Áreas Protegidas (RUNAP) 2024). To ensure confidentiality, we are not making original interview transcripts publicly available.
