# Supplementary material for: High-integrity forest carbon credits: Assessing equity and governance deficits in Colombia’s voluntary carbon market
Source: Ambio. 2026 Feb 25;55(8):1800–17. doi: 10.1007/s13280-025-02343-1 (PMC13319653; doi:10.1007/s13280-025-02343-1)
Supplement: Supplementary file 1 — Supplementary file1 (PDF 726 KB) [file 13280_2025_2343_MOESM1_ESM.pdf]

Supplementary Information: This Supplementary Information has not been peer reviewed.

Title: High-Integrity Forest Carbon Credits: Assessing Equity and Governance Deficits in Colombia's Voluntary Carbon Market

Methods

1. Spatial analysis of the carbon forestry market and projects in IPALC's territories Colombia

Spatial analysis was conducted and a set of indicators were calculated to assess the extent and regional distribution of CFP by type in Colombia; the extent and overlap by project type with IPALC's territories; the overlap with strategic ecosystems and protected areas; and the spatial distribution of projects with reported conflicts. The results of this analysis were visualized through a series of maps.

1.1. Carbon forestry projects database

We gathered information from four datasets and available Project Design Documents (PDDs), sourced from all five registries operating in Colombia: BioCarbon, COLCX, EcoRegistry (Cercarbono), Gold Standard, and Verra (VCS and CCBA), building on previous efforts to create a diagnostic of REDD+ in the Amazon (SINCHI, 2023) and the fair and equitable REDD+ agreements policy brief for the Amazon and the Pacific (WWF Colombia & CCAP, 2024). Registries compile datasets from information that developers upload to their platforms in order to register a project or conduct verifications at different stages. Additionally, we obtained datasets compiled by independent organizations such as Berkeley, Asocarbono, and WWF, which aggregate information from the registries and other secondary sources. With the consolidated dataset of 15,674 records, we identified 542 CFP categorized as REDD+, ARR, and/or AFOLU (Agriculture, Forestry, and Other Land Use) projects or similar designations for CFP specific for each dataset (Appendix A). Duplicates identified through project ID and name were eliminated, resulting in a dataset of **218 unique CFP project records (170 polygons and 48 points)**. The database was reviewed for completeness, and missing information was filled in using information from PDDs, where available. Variables such as project status and methodology protocol were standardized to create consistent categories across languages and similar concepts.

Geographic locations for each project were obtained from the original datasets as spatial data formats (shapefiles, geodatabase, or .kmz) or created from information provided in the PDD, and then classified into two geometries: polygons or points, prioritizing polygons where available. Overall, 12 polygons were created by georeferencing and vectorizing figures, while 47 points were derived from coordinates listed in the PDD. Errors in polygons, such as self-intersections or zero or negative areas were corrected using the Repair Geometry tools in ArcGIS Pro. The Pairwise Dissolve tool was then used to unify data by project ID, project type, and all available information, resulting in 3 distinct layers for analysis: the project ID

layer with **170 unique project polygons**, the project type layer with two polygons for REDD+ and ARR projects, and a single-polygon layer for all CFP. Both the project type and ID layers have overlaps. It should be noted that the results in terms of area coverage represent **170 out of 218 detected projects,** which account for those with exact project locations.

1.2. Market share, extent and regional distribution of carbon forestry projects

We analyzed the spatial extent of CFP by intersecting the single-polygon layer for all CFP with the country and regional boundaries of Colombia's five main regions to determine the total area of CFP relative to national and regional areas. Regional boundaries were sourced from the Departamento Administrativo Nacional de Estadistica (DANE) via their Geographic Data Portal on March 25, 2024 (DANE, 2018). To further analyze the extent and distribution by project type, we used the two-polygon layer to calculate areas covered by REDD+ or ARR projects. This information was visualized on a map (Figure 2).

To estimate potential monetary flows from carbon certificate transactions, we compared the total volume of issued credits to the volume of retired credits. The conversion of carbon tonnage into monetary value was based on the average price for land-use REDD+ projects reported by Forest Trends' Ecosystem Marketplace, which is USD 3.65 per certificate and considered a conservative value. Despite the higher prices reported in 2022 and 2023, a conservative carbon price was used in these estimates due to the recent decline in prices (Donofrio et al., 2019). Overall, monetary values in Colombian pesos were converted into US dollars with a rate of 4.325,05 USD/COP, the average conversion rate for 2023.

1.3. Extent and type of carbon forestry projects in IPALC's territories

We intersected the spatial extent of CFP (single polygon layer) and by type (two-polygon layer) with geographic data of recognized Indigenous Reserves (ANT, 2024b), Afro-descendant Councils (ANT, 2024a), and Peasant Reserves (ANT, 2024c) obtained from Agencia Nacional de Tierras (ANT) via their Open Data Portal on March 25, 2024. This resulted in a set of indicators on the areas and number of projects by type within IPALC's territories, as well as the areas of IPALC's territories without projects. The results were visualized on a map (Figure 3), displaying the overlap of REDD+/ARR projects with ILPC territories.

1.4. Strategic ecosystems overlap with carbon forestry projects and IPALC's territories

To analyze the extent of forested areas within IPALC's territories and CFP, we intersected the spatial extent of CFP by type (two-polygon layer) with IPALC's territories layers and the 2022 land surface forest cover (IDEAM, 2022). Additionally, we analyzed the overlap of CFP with Nationally Protected Areas, including the National Natural Parks system (RUNAP, 2024), and the map of continental, coastal and marine ecosystems (IDEAM, 2017) to determine the area of CFP per type of ecosystem.

1.5. Conflict analysis of carbon forestry projects in IPALC's territories

In order to assess the extent and spatial distribution of projects with reported conflicts in IPALC's territories, we created a dataset based on journalistic reports detailing conflicts between ethnic communities and project developers, most of which have on-going legal claims or processes ruled in favor of the communities, and classified the conflicts as community rights or emissions reductions. Community rights conflicts include cases where community rights have been violated or where participatory processes were not conducted according to the communities' governance procedures. Emissions reductions conflicts refer to projects where the claimed emissions reductions were overestimated. In the absence of a public conflict reporting mechanism or consolidated official data, media reports and particularly the investigative journalism of the Latin American Center for Investigative Journalism (CLIP) constitute the most comprehensive source of information on conflicts related to CFP. Neither the Ministry of Interior, responsible for guaranteeing IPALC rights, nor the Ministry of Environment, which regulates the carbon market, maintain such mechanisms, and the judiciary lacks a systematic consultation process to track related cases. As a result, official information remains fragmented and inaccessible, and conflicts are likely underreported. Nevertheless, CLIP's investigation, which includes project-level reviews, field visits, stakeholder interviews, provides a unique account of conflicts in rural and remote areas that would otherwise remain absent from national media coverage. Nevertheless, CLIP's investigation, which includes project-level reviews, field visits, stakeholder interviews, provides a unique account of conflicts in rural and remote areas that would otherwise remain absent from national media coverage. With the information consolidated by CLIP in the Anatomy of the Carbon Market platform, we identified 82 active projects in Colombia, from which 34 have reported conflicts. The review of additional media sources led to the identification of 4 more projects with reported conflicts and the overall identification of 37 projects within our consolidated database. Conflict data was integrated into the geodatabase, and the results were visualized in a map (Figure 4), displaying the overlap of projects with reported conflicts with IPALC's territories.

2. Qualitative analysis of the enabling and disabling factors for IPALC participation

We conducted a qualitative analysis process to advance the understanding of the enabling and disabling factors affecting IPALC effective participation in CFP within Colombia's VCM. This process involved semi-structured interviews, transcript coding, and the analysis of excerpts by coding categories according to six areas of interest associated with enabling and disabling factors, including **general context, regulatory framework**, **benefit sharing**, **governance and participation mechanisms**, existing **safeguards**, and the potential contributions of CFP to the Sustainable Development Goals (**SDGs**). Building on insights from the analysis, we developed a theoretical framework to assess IPALC engagement and proposed a roadmap for improving IPALC participation in VCMs, and applied it to the case of Colombia's VCM.

Semi-structured interviews: We conducted 20 interviews with stakeholders across the carbon certificate value chain to gain insight into the range of experiences related to the six areas of interest. Interviews were conducted in Spanish, virtually via Zoom. The criteria for selecting interviewees aimed to ensure diverse representation across sectors, and to capture both positive and negative experiences related to IPALC participation. The interviewees included five community members from Indigenous (2), and Afro-descendant (3) communities, representing different regions (1 Amazon; 3 Pacific; 1 Caribbean), and engaged in projects developed by international cooperation (1), and private developers (4); project developers (5), including the ones with the largest market share, and two with reported conflicts; and Validation and Verification Bodies (VVB) (2), Standards (1), and Registries (2) to cover all key stages of the value chain. Furthermore, (2) government stakeholders from the Ministry of Environment were selected based on their direct engagement with VCM regulation; and (5) experts from diverse fields, including academia (1), journalism (1), consulting (2) and VCM associations (1). It is important to note that some community members declined to participate for fear of drawing unwanted attention as leaders of CFP and potentially becoming targets for illegal groups, and that (4) of the largest carbon certificate buyers in Colombia declined to be interviewed.

Interview transcript coding: Data from the interviews was recorded and transcribed using Read AI software (with participants' consent) and analyzed through thematic coding (Appendix C) using Dedoose software following a three-step process. A set of first-level codes was created a priori to classify excerpts according to six areas of interest associated with enabling and disabling factors affecting IPALC participation, and second-level coding was applied, categorizing the excerpts as follows: **context** included 82 excerpts, categorized into enabling factors (35) and disabling factors (47); **regulatory framework** included 274 excerpts, categorized into challenges (170), best practices (36), achievements

(6), and recommendations (62); **benefit-sharing** mechanisms included 78 excerpts, categorized into challenges (20), best practices (38), and recommendations (20); **governance and participation** included 339 excerpts, categorized into challenges (127), best practices (160), and recommendations (52); **safeguards** included 60 excerpts, categorized into challenges (15), best practices (17), and recommendations (28); and **SDGs** included 82 excerpts, all categorized as best practices. Lastly, these categories were analyzed to identify the main concepts, and third-level codes were created a posteriori to reflect these concepts.

Framework for IPALC engagement: In order to assess the quality of IPALC engagement across Colombia's VCM and to propose a framework to assess IPALC engagement in VCMs, we segmented 287 **governance and participation** excerpts into 8 levels of participation. We analyzed the experiences associated with each level and used the findings to develop a theoretical framework for IPALC participation in VCMs. The framework builds on the literature of citizen participation and community engagement, specifically Arnstein's Ladder of Citizen Participation which uses steps that represent the stages of involvement of citizens in planning processes, ranging from non-participation at the lowest level, to full citizen control in planning at the highest level (Arnstein, 1969). Reconceptualized as the IPALC Participation Ladder for VCMs, our eight-level framework represents the challenges and opportunities faced by IPALC across different stages of participation in CFP in the VCM.

Appendix A - Consolidated data sources of carbon forestry projects

Source	Carbon forestry project designation	Nr of projects globally	Nr of projects in Colombia
VERRA	ARR-REDD	4180	55
ECOREGISTRY	Land use (AFOLU)	1884	65
COLCX	Forestación/Reforestación	46	36
BIOCARBON	Reduced emissions from deforestation & degradation, Forest Restoration, Reforestation	369	40
BERKELEY	A/R, Agriculture Forestry and Other Land Use	8777	57
ASOCARBONO	Aforestación/Reforestación, REDD+	332	203
WWF	AFOLU, REDD+ (Colombia)	86	86
Total			542
Total without duplicates			218

Appendix B - Projects with reported conflicts

Project	Community	Location	Type of reported conflict	Sources
Waldrettung - Resguardo Indígena Nukak Maku	Resguardo Indígena Nukak Maku	Guaviare	Impact on the Rights of Ethnic Communities: Shortcomings in the Processes of Free, Prior, and Informed Consent and Prior Consultation	https://es.mongabay.com/2022/09/indigenas-negocian-bonos-de-carbono-en-desventaja-y-sin-respaldo-estatal-en-colombia/
Pachamama Cumbal	Gran Resguardo de Cumbal	Nariño, frontera con Ecuador	Impact on the Rights of Ethnic Communities: Shortcomings in the Processes of Free, Prior, and Informed Consent and Prior Consultation	https://elpais.com/america-colombia/2023-09-19/un-juez-suspende-el-proyecto-de-carbono-en-colombia-hecho-de-espaldas-a-la-comunidad.html
Matavén	Resguardo Indígena de Selva de Matavén	Orinoquía y Amazonía	Overestimation of Emission Reductions and Environmental Impact	https://es.mongabay.com/2021/06/bonos-de-carbono-mataven-colombia-redd-investigacion/
Baka Rokarire	Resguardo Indígena Pirá Paraná	Vaupés	Impact on the Rights of Ethnic Communities: Shortcomings in the Processes of Free, Prior, and Informed Consent and Prior Consultation	https://www.infobae.com/colombia/2023/02/12/denuncian-corrupcion-en-la-negociacion-de-bono-carbono-de-mas-de-15000-millones-en-vaupes/<br><br>https://es.mongabay.com/2023/05/colombia-corte-constitucional-examinara-por-primera-vez-un-conflicto-por-bonos-de-carbono/
Proyecto Nonuya de Villazul	Resguardo de Nonuya de Villazul	Amazonas	Impact on the Rights of Ethnic Communities: Shortcomings in the Processes of Free, Prior, and Informed Consent and Prior Consultation	https://es.mongabay.com/2023/01/politico-sancionado-que-negocia-bonos-de-carbono-en-la-amazonia-colombia/
Banakale Isimali REDD+	Resguardos indígenas La Llanura	Vichada	Impact on the Rights of Ethnic Communities: Shortcomings in the Processes of Free, Prior, and Informed Consent and Prior Consultation	https://verdadabierta.com/con-aplausometro-biofix-logro-consentimiento-indigena-para-negociar-bonos-de-carbono/
Aire de Vida Fiivo Jaagava Komuya Jag+y+ Monochoa REDD+	Resguardo Indígena Monochoa	Caquetá - Amazonas	Impact on the Rights of Ethnic Communities: Shortcomings in the Processes of Free, Prior,	https://es.mongabay.com/2023/03/colombia-comunidades-indigenas-excluidas-de-un-proyecto-de-carbono-en-su-terr

			and Informed Consent and Prior Consultation	itorio/
Proyecto Kaliawiri	Resguardos de Cali-Barranquilla, Concordia, Flores Sombrero, Chocón, Río Siare Barranco Lindo, Saracure Río Cada, Guaco Alto Guaco Bajo	Vichada - Guania	Impact on the Rights of Ethnic Communities: Shortcomings in the Processes of Free, Prior, and Informed Consent and Prior Consultation	https://www.lasillavacia.com/silla-nacional/el-mayor-proyecto-de-bonos-de-carbono-de-colombia-podria-estar-vendiendo-aire-caliente/
Proyecto de Conservación Palameku Kuwei REDD+	Resguardo Ríos Muco y Guarrojo, Resguardo Ríos Tomo y Weberi, Resguardo Punta Bandera, Resguardo San Luis del Tomo, Resguardo La Esmeralda, Resguardo Valdivia	Vichada , Cumaribo	Impact on the Rights of Ethnic Communities: Shortcomings in the Processes of Free, Prior, and Informed Consent and Prior Consultation	https://infoamazonia.org/en/project/anatomy-of-the-carbon-market/
Proyecto de Conservación Delfines Cupica REDD+	Consejo Comunitario General Costa Pacífica Norte del Chocó - Los Delfines, Consejo Comunitario Cupica	Chocó, Bahia Solano	Impact on the Rights of Ethnic Communities: Shortcomings in the Processes of Free, Prior, and Informed Consent and Prior Consultation	https://infoamazonia.org/en/project/anatomy-of-the-carbon-market/
Proyecto de compensación de emisiones Conservación del Bosque Galilea-Amé	212 private properties	Cundinamarca, Tolima	Impact on the Rights of Ethnic Communities: Shortcomings in the Processes of Free, Prior, and Informed Consent and Prior Consultation	https://www.elclip.org/bonos-carbono-bosque-galilea/
Proyecto de Mitigación Forestal Resguardo Indígena Tikuna, Cocama y Yagua (Ticoya)	Resguardo Indígena Tikuna, Cocama y Yagua (Ticoya)	Amazonas Leticia	Impact on the Rights of Ethnic Communities: Shortcomings in the Processes of Free, Prior, and Informed Consent and Prior Consultation	https://www.elclip.org/bosque-galilea-campesinos-tierra-colombia/

Proyecto de Conservación Tángara REDD+	2 private properties	Valle del Cauca Buenaventura	Impact on the Rights of Ethnic Communities: Shortcomings in the Processes of Free, Prior, and Informed Consent and Prior Consultation	https://infoamazonia.org/en/project/anatomy-of-the-carbon-market/
REDD+ Dabucury	Resguardo Lagos El Dorado, Lagos del Paso y El Remanso, Resguardo Vuelta del Alivio, Resguardo Yavilla II	Guaviare	Impact on the Rights of Ethnic Communities: Shortcomings in the Processes of Free, Prior, and Informed Consent and Prior Consultation	https://infoamazonia.org/2023/10/24/empresas-colombianas-ignoram-funai-e-leis-brasileiras-em-projetos-de-carbono-na-amazonia/
REDD+ El Tigre	Resguardo Guahibo de la Región de El Tigre	Meta, Puerto Gaitán	Impact on the Rights of Ethnic Communities: Shortcomings in the Processes of Free, Prior, and Informed Consent and Prior Consultation	https://www.elclip.org/indigenas-monochoa-exclusion-bonos-carbono/
Proyecto de Conservación Arlequín REDD+	Consejo Comunitario Integral de Lloró (Cocoillo), Consejo Comunitario Mayor de Cértegui (Cocomacer), Consejo Comunitario Mayor de Cantón de San Pablo (Acisanp)	Chocó	Impact on the Rights of Ethnic Communities: Shortcomings in the Processes of Free, Prior, and Informed Consent and Prior Consultation	https://infoamazonia.org/en/project/anatomy-of-the-carbon-market/
Manejo Sostenible de los Bosques Aplicado en el Oriente Antioqueño bajo el esquema BanCO2	149 private properties	Antioquia, Argelia, Cocorná, El Carmen de Viboral, Guatapé, Nariño,	Impact on the Rights of Ethnic Communities: Shortcomings in the Processes of Free, Prior, and Informed Consent and Prior Consultation	https://www.elclip.org/bonos-carbono-yurupari/
Proyecto REDD+ Conservando la Vida del Mundo, Mowíchina arü Maü, Ríos Cotuhe y Putumayo	Resguardo Ríos Cotuhe y Putumayo	Amazonas, Leticia, Puerto Nariño, Tarapacá	Impact on the Rights of Ethnic Communities: Shortcomings in the Processes of Free, Prior, and Informed Consent and Prior Consultation	https://www.elclip.org/chevron-compro-bonos-de-carbono-a-proyecto-hecho-nuevamente-de-espaldas-a-indigenas/

Proyecto CRIMA Predio Putumayo y Andoque de Aduche REDD+	Resguardo Andoque de Aduche, Resguardo Predio Putumayo	Amazonas, Caquetá, La Chorrera, Puerto Alegría, Puerto Santander, Solano	Impact on the Rights of Ethnic Communities: Shortcomings in the Processes of Free, Prior, and Informed Consent and Prior Consultation	https://infoamazonia.org/en/project/anatomy-of-the-carbon-market/
Putumayo REDD+	Resguardo Alto Orito, Resguardo Simorna, Resguardo Inga de San Andrés, Resguardo Nukanchipa Alpa Amukunapa Wasi	Amazona, Andes, Santiago, Villagarzón, Orito, San Francisco, Pasto	Impact on the Rights of Ethnic Communities: Shortcomings in the Processes of Free, Prior, and Informed Consent and Prior Consultation	https://infoamazonia.org/en/project/anatomy-of-the-carbon-market/
Proyecto REDD+ de los pueblos indígenas del Vaupés Yutucu y Otros	Kubeo, Wanano, Barasano, Tatuyo, Tuyuca, Piratapuyo, Desano	Vaupes, Caruru, Mitú, Papunaua, Yavaraté	Impact on the Rights of Ethnic Communities: Shortcomings in the Processes of Free, Prior, and Informed Consent and Prior Consultation	https://www.elclip.org/bonos-carbono-bosque-galilea/
REDD+ Marena Ichena - Nag+ma Enoye Rafue	Muina Murui (Uitoto)	Caqueta, Solano	Impact on the Rights of Ethnic Communities: Shortcomings in the Processes of Free, Prior, and Informed Consent and Prior Consultation	https://infoamazonia.org/en/project/anatomy-of-the-carbon-market/
Makaro Ap+ro	Kubeo, Wanano, Desano	Vaupés, Mitú	Impact on the Rights of Ethnic Communities: Shortcomings in the Processes of Free, Prior, and Informed Consent and Prior Consultation	https://infoamazonia.org/en/project/anatomy-of-the-carbon-market/
Awakadaa Matsiadali	Curripako, Puinave, Sikuani, Piapoco	Guainía, Barrancominas, Inírida	Impact on the Rights of Ethnic Communities: Shortcomings in the Processes of Free, Prior, and Informed Consent and Prior Consultation	https://www.elclip.org/amonestacion-corte-constitucional-empresas-carbono-y-gobierno-colombiano/
Jocū Bucūrō Apūrō	Wanano, Piratapuyo, Tukano	Guaviare, Miraflores	Impact on the Rights of Ethnic Communities: Shortcomings in the Processes of Free, Prior, and Informed Consent and Prior Consultation	https://www.elclip.org/delta-airlines-bonos-carbono-proyecto-problemas/

Embera REDD+	Embera	Chocó, Bojayá, Riosucio	Impact on the Rights of Ethnic Communities: Shortcomings in the Processes of Free, Prior, and Informed Consent and Prior Consultation	https://www.elclip.org/indigenas-monochoa-exclusion-bonos-carbono/
Proyecto de Conservación Cocoman Frontera REDD+	Consejo Comunitario Mayor de Juradó, Consejo Comunitario Mayor de Nóvita (Cocoman)	Chocó, Juradó, Novita	Impact on the Rights of Ethnic Communities: Shortcomings in the Processes of Free, Prior, and Informed Consent and Prior Consultation	https://infoamazonia.org/en/project/anatomy-of-the-carbon-market/
REDD+ Nunuya de Villa Azul Amenanae	Muinane, Nonuya	Amazonas, Caquetá, Mirití-Paraná, Solano	Impact on the Rights of Ethnic Communities: Shortcomings in the Processes of Free, Prior, and Informed Consent and Prior Consultation	https://www.elclip.org/politico-bonos-de-carbono-amazonia-colombia/
Pedeguita y Mancilla REDD+	Consejo Comunitario Pedeguita-Mancilla	Chocó, Belén de Bajirá, Carmen del Darién, Riosucio	Impact on the Rights of Ethnic Communities: Shortcomings in the Processes of Free, Prior, and Informed Consent and Prior Consultation	https://infoamazonia.org/en/project/anatomy-of-the-carbon-market/
Pitugucajude	Piratapuyo, Tukano, Desano	Vaupés, Yavaraté	Impact on the Rights of Ethnic Communities: Shortcomings in the Processes of Free, Prior, and Informed Consent and Prior Consultation	https://www.elclip.org/amonestacion-corte-constitucional-empresas-carbono-y-gobierno-colombiano/
Proyecto de Conservación Yaawi Iipana REDD+	Puinave, Curripako, Nukak, Kubeo	Guaviare, El Retorno, San José del Guaviare	Impact on the Rights of Ethnic Communities: Shortcomings in the Processes of Free, Prior, and Informed Consent and Prior Consultation	https://www.elclip.org/el-proyecto-de-carbono-en-el-pacifico-colombiano-que-se-traslapa-con-territorios-afro/
Proyecto de Conservación Chonta Corozo REDD+	Consejo Comunitario de Alto Guapi	Cauca, Guapi	Impact on the Rights of Ethnic Communities: Shortcomings in the Processes of Free, Prior, and Informed Consent and Prior Consultation	https://www.elclip.org/el-proyecto-de-carbono-en-el-pacifico-colombiano-que-se-traslapa-con-territorios-afro/
Proyecto de Conservación Unu-Mai REDD+	Resguardo Laguna Niñal, Cocuy, Loma Baja y Loma Alta del Caño	Guainía, Inírida	Impact on the Rights of Ethnic Communities: Shortcomings in the Processes of Free, Prior, and Informed Consent and	https://www.elclip.org/el-proyecto-de-carbono-en-el-pacifico-colombiano-que-se-traslapa-con-territorios-afro/

	Guariben		Prior Consultation	
Corredor de Robles Guantiva-La Rusia-Iguaque	153 private properties	Santander, Coromoro, Encino, Charalá, Onzaga, Mogotes, Gámbita	Impact on the Rights of Ethnic Communities: Shortcomings in the Processes of Free, Prior, and Informed Consent and Prior Consultation	https://www.elclip.org/bosque-galilea-campesinos-tierra-colombia/
Pago por servicios ambientales del esquema de manejo forestal y conservación de los recursos hídricos en la Jurisdicción de Corpochivor (ERSA)	Undetermined number of private properties	Boyacá	Impact on the Rights of Ethnic Communities: Shortcomings in the Processes of Free, Prior, and Informed Consent and Prior Consultation	https://infoamazonia.org/en/project/anatomy-of-the-carbon-market/
Proyecto de Carbono Azul Golfo de Morrosquillo ""Vida Manglar""		Córdoba, Sucre	Impact on the Rights of Ethnic Communities: Shortcomings in the Processes of Free, Prior, and Informed Consent and Prior Consultation	https://infoamazonia.org/en/project/anatomy-of-the-carbon-market/
Proyecto REDD+ Magnolios de Yarumal	125 private properties	Antioquia, Briceño, Santa Rosa de Osos, Valdivia, Yarumal	Impact on the Rights of Ethnic Communities: Shortcomings in the Processes of Free, Prior, and Informed Consent and Prior Consultation	https://infoamazonia.org/en/project/anatomy-of-the-carbon-market/
Condoto REDD+	Consejo Comunitario Mayor de Condoto-Iró	Chocó, Condoto	Impact on the Rights of Ethnic Communities: Shortcomings in the Processes of Free, Prior, and Informed Consent and Prior Consultation	https://infoamazonia.org/en/project/anatomy-of-the-carbon-market/

Appendix C - Summary of interview insights

REGULATORY FRAMEWORK		
**Challenges and Limitations**	**Best Practices**	**Recommendations**
Market failure: *Systemic market failures occur throughout the market chain due to information*	*Some stakeholders have proactively addressed information asymmetry by ensuring a thorough*	

asymmetry, conflicts of interest, lack of transparency in transactions, the emergence of low-quality stakeholders and projects driven by profit maximization, and lack of alignment with the NDC.	*pre-project preparation phase, developing tools to verify overlaps, including special contract clauses to keep the community informed about future sales prices, and having their own legal teams.*	
**a) Lack of access and transparency** to project-related information, such as benefit-sharing, sales and resale prices, investment costs, and funding sources, leaves IPALC without the information to differentiate between developers' quality, leading to suboptimal selection.<br><br>**b) Difficulty in identifying overlaps** due to the lack of an operational emissions reduction registration system results in communities sometimes agreeing to projects for the same territory.<br><br>**c) Sale transactions lack traceability,** limiting capacities to detect non-compliance with contract clauses for benefit-sharing.<br><br>**d) Demonstrate additionality** in projects because some communities receive certificates for activities preceding project development or projects are developed in areas not at risk of deforestation.<br><br>**e) Contribution to the national emission reduction goal:** The absence of regulations establishing a minimum percentage of reductions aligned with Colombia's NDCs could mean that projects do not contribute to the national emission reduction goal.<br><br>**f) Supply-side failure:** abundance of low-quality auditors and certifiers who do not detect problems in the territory and who publish incomplete information, developers and communities not	**a) Pre-project preparation process conducted by international development agencies** to strengthen governance practices, knowledge transfer in VCMs, seed funding, and community support.<br><br>**b) A contract clause regarding sale prices** is now demanded as mandatory by one Indigenous community to remain informed about credit's sale prices and secure participation in re-sale transactions.<br><br>**c) Tools to verify overlaps** have been developed by standards and VVBs to cover RENARE's inoperability.<br><br>**d)** Some VVBs have used **representative samples of the community** to assess their understanding of the project when a comprehensive auditing process is hindered by the challenges posed by extensive territory or violence.<br><br>**e)** Community events, such as cultural festivities, have been used as **complementary spaces to socialize information about REDD+ projects** due to the high attendance of community members.<br><br>**f) Some communities have their own legal team** that internally reviews all documentation and contracts related to the REDD+ project.<br><br>**g)** A community decided to **sell carbon certificates from its projects only to organizations**	**Government:**<br>**a) Regulate the minimum conditions for operating as a developer or VVB** by establishing the technical and financial capacities necessary to implement or verify carbon forestry projects and creating a conflict-of-interest policy, potentially through the proposed Environmental Regulatory Commission.<br><br>**b) Create an environmental superintendence** that exercises the functions of inspection, surveillance, and control of carbon market actors, with legal powers to impose sanctions on those who violate the reference frameworks on deforestation baselines, compliance with safeguards, and sales reports.<br><br>**c) Strengthen the role of the regional autonomous corporations** in providing technical assistance to IPALC participating in the carbon market by supporting communities in negotiation processes and providing training on their rights, environmental management practices, and financial practices.<br><br>**d) Create a unified information system for registering emissions reduction projects**, with advanced technology to detect overlapping projects in the same territory and record sales transactions (possibly including technologies like blockchain to provide a secure, transparent, and decentralized way to record and verify transactions).<br><br>**IPALC:**<br>**a) Introducing a contract clause**

transparent about project overlaps, developers forging procedures and documentation to obtain project approvals and secure bond issuances, among others.<br><br>**g) Demand-side failure:** companies buying carbon bonds without conducting any due diligence.<br><br>**h) Conflict of interest:** Different actors fulfill more than one role in the chain. For example, buyers have been reported to act as certifiers, owners of the standard are owners of the company that buys the bonds, developers hire former partners as auditors, etc.	**and companies demonstrating additional efforts to reduce carbon emissions.**	**that ensures communities receive benefits from future resales** will promote more equitable benefit-sharing for communities; nonetheless, further transparency and accountability in the entire transaction process are necessary to verify compliance.<br><br>**b) Establish accountability assemblies and community oversight groups** where ethnic authorities periodically report to the community on the use and management of economic resources generated by the projects.<br><br>**Developers:**<br>**a) Introducing a contract clause that ensures communities receive benefits from future resales** will promote more equitable benefit-sharing for communities; nonetheless, further transparency and accountability in the entire transaction process are necessary to verify compliance.<br><br>**VVBs:**<br>**a) VVB's verification mechanisms** should include a combination of documental and field-work processes, without exception.
Institutional capacities: *The state has limited capacities necessary to ensure the effective functioning of carbon markets, including the operation of information systems, oversight and sanctioning capacities, technical support for IPALC, and the implementation of prior consultation processes.*	*The Ministry of Environment has increased community support, and some VVBs have created tools to compensate for the lack of a unified information platform. These changes coincide with a shift in the government's approach to carbon markets from a flexible market-oriented perspective to a human rights issue.*	
**a) Lack of an operational and unified environmental platform** that integrates all records required by various regulations, including registering emission reduction projects.<br><br>**b) Lack of technological**	**a) The Ministry of Environment has started to provide greater support to communities**, including online training modules and educational materials.<br><br>**b) Tools to verify overlaps** have been developed by standards and	**Government:**<br>**a) Create a unified information system** for the registration of emissions reduction projects, with advanced technology to detect overlap of projects in the same territory and record sales transactions (possibly including

**capacities** to develop a robust information system for emissions accounting with the capacity to detect overlaps between projects and track sales and re-sales transactions.<br><br>**c) Limited outreach and technical support** from government agencies to communities to facilitate their understanding of the carbon market, safeguards, and the communities' rights in the projects, and a lack of incentives for developers to transfer these capacities.<br><br>**d) Lack of an institution in charge of oversight, auditing, and enforcement of penalties** for VVB and their auditors, standards, and developers.<br><br>**e) Government staff** from agencies in charge of protecting community rights **lack knowledge** of carbon markets to effectively **advocate for IPALC' rights** within carbon forestry projects.<br><br>**f) Insufficient government resources for prior consultation processes** to ensure widespread prior consultations, leading developers to take on the costs of prior consultation.	VVBs to compensate for RENARE's inoperability.	technologies like blockchain to provide a secure, transparent, and decentralized way to record and verify transactions)<br><br>**b) Strengthen the role of the regional autonomous corporations** in providing technical assistance to IPALC participating in the carbon market by supporting communities in negotiation processes and providing training on their rights, environmental management practices, and financial practices.<br><br>**c) Create an environmental superintendence** that exercises the functions of inspection, surveillance, and control of carbon market actors, with legal powers to impose sanctions on those who violate the reference frameworks on deforestation baselines, compliance with safeguards, and sales reports.<br><br>**IPALC:**<br>a) **Establish criteria in their internal norms** to determine when the community considers prior consultation necessary or an alternative free and informed consent process to guarantee the participation of most community members in the decision-making process.
Perverse incentives:<br>*The lack of uniformity and regulation for national and international standards allows developers to arbitrarily select baseline scenarios, select standards and VVBs that create fewer barriers, and generally choose the most convenient options.*	***Investment models without non-profit intermediaries and check and balances systems*** *minimize the perverse incentives associated with profit-driven intermediaries.*	
**a) Unfair competition among actors** sacrifices quality to maximize profits, attract and maintain customers, and approve	**a) International cooperation agencies developed resource management mechanisms centered around a revolving**	**Government:**<br>**a) Establish an environmental regulatory commission** to issue rules on the frame of reference for

projects more easily and quickly.<br><br>**b) Arbitrary selection of deforestation baselines** allows project developers to choose the most favorable baselines despite projects related to the national carbon tax requiring the use of the National Reference Scenario.<br><br>**c) The highly flexible scenario, created on purpose to incentivize the growth of the carbon market,** favors private actors' interests. Nonetheless, weaknesses in control allow intermediaries to take advantage to the detriment of communities.<br><br>**d) Complexity and technical language barriers benefit developers**, but they also limit the capacities of IPALC to be informed and effectively negotiate the terms of engagement.<br><br>**e) The Lack of homogeneity among standards** allows for the emergence of VVBs and developers with varied capacities. This, combined with profit-driven purposes, has resulted in the prevalence of low-quality VVBs and developers.<br><br>**f) Methodologies are becoming less rigorous**, allowing anyone to create them. Standards organizations should refrain from developing their own methodologies.	**fund** (fondo rotatorio) that enables communities to maximize their participation in carbon forestry projects by providing initial investments and eliminating the need for profit-driven intermediaries.<br><br>**b) One standard is undergoing a comprehensive review of most of its methodologies through an independent third party** (independent experts) to provide feedback and strengthen its methodologies based on current good practices.<br><br>**c) An independent third party within VVB** whose main task is to audit the project and oversee the work of VVB's auditors. Essentially, this person functions as an internal auditor who monitors the performance of others.	defining deforestation baselines, quality criteria for carbon certificates, sales price tracking, and compliance with environmental and social safeguards.<br><br>**b) Create an environmental superintendence** that exercises the functions of inspection, surveillance, and control of carbon market actors, with legal powers to impose sanctions on those who violate the reference frameworks on deforestation baselines, compliance with safeguards, and sales reports.<br><br>**c) Create a certifying agency for developers, standards, and VVB** that establishes conditions for operation and grants licenses, overseeing the actors' technical and financial capacities.<br><br>**d) Regulate the minimum conditions for operating as a developer or VVB** by establishing the technical and financial capacities necessary to implement or verify voluntary carbon forestry projects and creating a conflict-of-interest policy, potentially through the proposed Environmental Regulatory Commission.
		**VVBs:**<br>**a) Adopt an internal auditor** to oversee the work of other auditors who can identify low performance, disseminate best internal practices, and set higher standards.
		**Registries and Standards:**<br>**a) Adopt a protocol to review methodologies periodically through an independent third party** that can provide recommendations to strengthen the methodologies based on current best practices.

BENEFIT-SHARING		
**Challenges and Limitations**	**Best Practices**	**Recommendations**
Lack of an associated regulatory framework to the safeguard related to benefit distribution.	*Some stakeholders, including VVBs, international cooperation, and communities, have taken proactive measures to compensate for regulatory gaps, including nudges, full ownership agreements, land-based distribution, and the engagement of the ombudsman.*	
**a)** The safeguard related to the benefit-sharing **does not have an associated regulatory framework.**<br><br>**b)** Despite having the best comparative information, **VVBs lack the basis for making recommendations about benefit-sharing equity.**<br><br>**c)** Because of **information asymmetries**, communities are unaware they can improve project benefit-sharing.	**a) Nudges from VVBs** in the form of inquiries aimed at raising awareness in the community about equity in benefit-sharing.<br><br>**b) Full ownership by the community of the project's credits and 99% of the economic benefits.** Projects developed with international cooperation exemplify community empowerment, where IPALC entirely own credits, receiving 99% of the financial benefits.<br><br>**c) Land-based benefit-sharing** when there is more than one community involved who owns different extensions of land<br><br>**d) A code of ethics** developed between the developer and the National Association of indigenous communities to determine the minimum benefit-sharing percentage for communities working with developers accounting for territories of diverse extensions.<br><br>**e) Convening the public ministry to community assemblies** that discuss benefit-sharing as a guarantor of community rights.	**Government:**<br>**a) Develop a regulatory framework for the safeguard regarding benefit-sharing to ensure equitable benefit allocation**, determining a minimum percentage of benefit-sharing for communities that considers the size of the territories, a dynamic distribution that decreases over the lifetime of a project in relationship with the investment, and the minimums established in the code of ethics developed by the Indigenous Association OPIAC in collaboration with project developers, which considers a minimum of no less than 60% for territories of more than one million hectares, and 75% for smaller territories.<br><br>**IPALC:**<br>**a) Establish minimum requirements for participation** in carbon forestry projects associated with equitable benefit-sharing between communities and developers in their life manuals or internal norms. Consider that the first years of implementation usually have higher costs, but as those decrease over time, so should the developers' percentage.<br><br>**b) Negotiate benefit-sharing percentages based on total sale value**, not revenue.<br><br>**VVBs:**<br>**a)** Lacking a national standard for

		minimum benefit-sharing to report unfair benefit-sharing in carbon projects, consider the **ethics code produced by IPALC associations as grounds for making recommendations or reporting unfair benefit-sharing.**<br><br>**Registries and Standards:**<br>**a) Benefit-sharing should be public data** to increase transparency and accountability.
Lack of transparency in sale and resale transactions hinders the assessment and verification of benefit-sharing.	*At least one standard and some communities have taken proactive measures to compensate for the lack of transparency in the benefit-sharing process, including benefit-sharing agreements on final sale values and developing a tool for financial information disclosure.*	
**a) The Lack of transparency in sell prices prevents verification of** whether communities receive a lower percentage of the final sale value than stipulated in the contract with the developer.<br><br>**b) Confidentiality restrictions** prevent VVBs from evaluating or reporting benefit-sharing concerns	**a) Benefit-sharing on final sale value** so that if a broker achieves a higher price for the bonds, the communities also receive part of that benefit and not only from the initial sale.<br><br>**b) A tool for financial information disclosure** is being developed by a standard. The tool, which will be voluntary, encourages the developer to include all the financial information of a project so that the benefit-sharing can be more transparent.	**Government:**<br>**a) Create a mandatory report of carbon credit transactions**, including at least information about the sellers, buyers, and value. Explore the potential of technologies like blockchain to provide a secure, transparent, and decentralized way to record and verify transactions.<br><br>**IPALC:**<br>**a) Introducing a contract clause that ensures communities receive benefits from future resales** will promote more equitable benefit-sharing for communities; nonetheless, further transparency and accountability in the entire transaction process are necessary to verify compliance.<br><br>**Registries and Standards:**<br>**a) Widely adopt emerging financial traceability mechanisms** to guarantee transparency in sale and resale transactions and make them mandatory.
Varied and high costs of required actions for the start of the project,	*A model stewarded by International Cooperation in the*	

verification, and validation act as entry barriers to the market for communities to be their own developers and influence benefit-sharing associated with the need for intermediaries.	*Pacific region has allowed the communities to address entry barriers associated with the initial costs of implementation and verification while guaranteeing full ownership of projects for the communities.*	
**a) Entry costs associated with implementing required actions at the start of a project vary significantly** and are not necessarily correlated with the percentage charged by developers, which often oscillates around 30%.<br><br>**b) Increased technical knowledge in the community** could decrease the cost of project development but not the costs associated with validation and verification or implementation costs.	**a) International Cooperation resources** have proven effective in eliminating entry barriers for communities, resulting in projects where the communities own all the carbon certificates, and only 1% is charged by an international cooperation agency for project administration.	**Government:**<br>a) **Channel international cooperation resources** to maximize community benefits by reducing the need for intermediaries who capture a percentage of the benefits that otherwise would go to the communities.<br><br>**b) Provide technical assistance and access to finance** to IPALC to facilitate their understanding of the carbon market, strengthen their capacities in project formulation, and transition to becoming project developers.
Without robust governance mechanisms, transparent financial practices, and financial education, the economic resources from carbon forestry projects can generate territorial fracture and problematic benefit-sharing within the community.	*Some developers and communities have adopted proactive measures to ensure transparency and effective resource management, such as trust funds, accountability committees, provision of administrative support, and alignment with communities' life plans.*	
**a) Carbon forestry projects introduce significant financial resources to communities, generating fragmentation in governance structures** when resources do not enter the territory through its authorities. This sudden influx can sometimes be misused to sway community leaders in favor of the project's development.<br><br>**b) Some communities report not having access to essential project documentation and information** on how project resources were spent.<br><br>**c) The extensive use of cash for**	**a) Investment priorities aligned with communities' life plans** to guarantee that resources are appropriately allocated to local and agreed priorities<br><br>**b) Trust funds with specific lines of investment and mandatory proof of expenditure** before releasing the funds are commonly used by developers as a mechanism to guarantee transparency and alignment in the use of resources with previously agreed-upon priorities.<br><br>**c) Comprehensive resources administration to strengthen the community's** financial and project	**Government:**<br>**a) Strengthen government programs** (Ministry of the Interior) that promote training within the IPALC on the governance mechanisms.<br><br>**b) Support the creation of community oversight groups** within the IPALC to monitor its authorities' administration of community resources.<br><br>**IPALC:**<br>**a) Establish accountability assemblies and community oversight groups** where ethnic authorities periodically report to the community on the use and

**community transactions hinders transparency and traceability** in resource use and is prone to corruption.<br><br>**d) Weaknesses in local capacities** to track resource use hinder the proper allocation of resources for priority projects.	management competencies in goal setting and project monitoring.<br><br>**d) Accountability committees and community assemblies** that oversee developers' resource management and ensure transparent community use.	management of economic resources generated by the projects.<br><br>**b) Create yearly work plans** to invest resources from carbon forestry projects on actions to advance the life plans and present these plans for approval in community assemblies.
		**Developers:**<br>**a) Promote the creation of community oversight groups** to monitor the project resources invested by ethnic authorities in the activities defined and agreed upon by the majority of the community.<br><br>**b) Adopt the practice of creating trust funds** with specific investment lines and mandatory proof of expenditure before releasing the funds to guarantee transparency and alignment in the use of resources with previously agreed-upon priorities.
		**VVBs:**<br>**a) Verify the existence of community oversight groups and accountability assemblies** that increase transparency in the use and management of resources.

**SAFEGUARDS**		
**Challenges and Limitations**	**Best Practices**	**Recommendations**
Safeguards are not legally binding and do not cover all aspects of carbon markets, are affected by a lack of coordination in compliance control, and compliance reports are not being publicly disclosed.	*Some stakeholders, including VVBs, standards, and communities, have taken proactive measures to compensate for the lack of regulation. These include developing a tracking tool or incorporation into a standard, VVBs' social impact evaluations, and safeguards in community contracts. The government has expanded the National Safeguards system to the AFOLU sector and incorporated safeguards into the*	

	National Development Plan.	
**a)** Colombia has a **national safeguards interpretation**, initially intended for the Warsaw framework of REDD+ and now used across the AFOLU projects sector. This interpretation is **not legally binding** and does not cover all aspects of VCMs.<br><br>**b) Existing regulations do not address how to comply with safeguards**, and due to the complexity of the issues that safeguards address, there is a lack of clarity and coordination regarding which agencies are responsible for controlling compliance.<br><br>**c) The lack of a centralized system like RENARE** allows actors to refrain from making safeguards compliance reports public.	**a) Tracking tool** for real-time tracking and monitoring of safeguards under development by a standard without a generalized and mandatory tool.<br><br>**b) Social impact evaluations** are conducted by VVBs to identify and mitigate social impacts connected to safeguards such as maintaining cultural traditions.<br><br>**c) Safeguards compliance** incorporation into standards and VVBs' verification process, such as scrutinizing PDDs.<br><br>**d) Article 185 of the National Development Plan** states that environmental and social safeguards are mandatory and need to be regulated, expanding the coverage of the National Safeguards System from REDD+ to the entire AFOLU sector.<br><br>**e) Safeguards incorporation into contracts** between the community and the developers to guarantee community rights.	**Government:**<br>**a) Regulate article 185, paragraph 2 of the National Development Plan 2022-26 to update the National System of Environmental and Social Safeguards**. The regulation needs to establish the mandatory basis for carbon forestry projects, define the competencies of government authorities to conduct outreach, determine indicators, supervise and enforce compliance, operate a public information system for the periodic reporting and monitoring of safeguard compliance, and emit alerts in the event of non-compliance.<br><br>**b) Create an environmental superintendence** that exercises the functions of inspection, surveillance, and control of carbon market actors, with legal powers to impose sanctions on those who violate the reference frameworks on deforestation baselines, compliance with safeguards, and sales reports.<br><br>**IPALC:**<br>**a) A contract clause should be introduced to adopt the specific safeguards** on each project and report any issues with their compliance to the Ministry and the association's ethics committee.<br><br>**b) Establish safeguards assemblies and community oversight groups** where ethnic authorities and developers periodically report to the community on safeguard compliance.
There is a systemic failure in safeguards related to social aspects, and safeguards fail to represent community interests.	*Some stakeholders have taken proactive measures to address the challenges associated with the social aspects of safeguards, such as conducting workshops with communities, establishing ethics committees, and creating fluid*	

	communication channels with government agencies.	
**a) The vast majority of projects fail in four interrelated safeguards**: broad community participation, access to information about the project, accountability for results, and free prior informed consent.<br><br>**b) Developers are good in the technical aspects but deficient in the social aspects** of projects because they lack an understanding of the Indigenous worldview.<br><br>**c) Safeguards were created under the vision of external actors** who carry out REDD+ projects and fail to incorporate the community perspective.<br><br>**d) The Cancun safeguards are too general,** so they need interpretation frameworks not only at the national level but also at the community level.	**a) Developers conduct workshops with communities** on the topic of safeguards.<br><br>**b) Developers verify with local government agencies** to guarantee that they are aware of the project's development.<br><br>**c)** The sector association, Asocarbono, implemented **an evaluation and ethics committee** that conducts relevant investigations if a community presents a complaint regarding a project.<br><br>**d)** The association for the sector, Asocarbono, **reviewed safeguards compliance to improve methodologies**.<br><br>**e) A community adopted safeguards related to maintaining cultural values** and the territory's cultural and environmental heritage and strengthening community governance (participation), among other things.	**Government:**<br>**a) The government must regulate Article 185, paragraph 2 of the National Development Plan 2022-26, protecting communities' right to self-determination** regarding social safeguards and allowing them to define how to implement each social safeguard in their territory.<br><br>**IPALC:**<br>**a) Define rules to implement social safeguards within the territories** and incorporate compliance procedures and definitions in their life manuals or internal norms<br><br>**Developers:**<br>**a) Train developer's work teams to understand the cosmovision and culture of IPALC** and how to comply with social safeguards according to the community's vision.<br><br>**b)** In the absence of a national system to report safeguard compliance, **select standards that make safeguards compliance reports public.**<br><br>**VVBs:**<br>**a) Guarantee safeguards' review in the validation and verification process** that considers the definitions and compliance processes of the social safeguards defined by specific communities.

GOVERNANCE AND PARTICIPATION		
**Challenges and Limitations**	**Best Practices**	**Recommendations**
There is unequal negotiating power between developers and IPALC, given significant entry barriers, information asymmetry,	*Multiple actors have developed strategies to increase communities' negotiation leverage, including technical assistance and*	

<table>
<tr><td>and limited technical expertise and institutional support.</td><td>education, access to financial resources, codes of ethics, and third-party advisors.</td><td></td></tr>
<tr><td rowspan="3">a) Developers, as project designers and sellers of the carbon certificates, are in a stronger position when negotiating with communities.

b) Technical expertise, language barriers, and the volume of projects under development give the developers of carbon projects an information advantage in negotiating with communities.

c) Acute unsatisfied basic needs pressure IPALC to prioritize expediency in the negotiation.

d) During the negotiating process, communities have limited Institutional support and must rely mostly on information provided directly by the developer, which may result in unequal leverage.

e) REDD+ projects have high investment costs, which the developers often fund. These costs act as an entry barrier for communities to govern the project</td><td rowspan="3">a) The Ministry of Environment has started to provide greater support to communities, including online training modules and educational materials.

b) Pre-project preparation process conducted by international development agencies to strengthen governance practices, carbon markets knowledge transfer, seed funding, and community support.

c) Nudges from VVBs in the form of inquiries to raise community awareness about fair benefit sharing.

d) A code of ethics developed between the developer and the National Association of Indigenous Communities (ONIC) to determine the minimum benefit-sharing for communities working with developers accounting for territories of diverse extensions

e) Some developers provide third-party experts selected by the community to advise the community during the project development phase.

f) International Cooperation has offered support to overcome entry barriers for communities by providing the financial capacity and technical support to launch the project with full ownership.</td><td>Government:
a) Strengthen the role of the regional autonomous corporations in providing technical assistance to IPALC participating in the carbon market by supporting communities in negotiation processes and providing training on their rights, environmental management practices, and financial practices.

b) Channel international cooperation resources to maximize community benefits by reducing the need for intermediaries who capture a percentage of the benefits that otherwise would go to the communities.

c) Provide technical assistance and access to finance to IPALC to facilitate their understanding of the carbon market, strengthen their capacities in project formulation, and transition to becoming project developers.</td></tr>
<tr><td>IPALC:
a) In the absence of technical assistance provided by government agencies, the developer should be required to hire independent technical experts selected by the community to provide advice on the project's development and negotiation phase.

b) Ensure that members (internal or external advisors) who represent the community's interests in negotiations have the technical capacities to understand a project's cost structure, the financial costs of investments, projected income and profits, knowledge of the market for the placement of credits, etc.</td></tr>
<tr><td>Developers:</td></tr>
</table>

		**a)** In the absence of technical assistance provided by government agencies, **support experts selected by the community** who certify they do not have conflicts of interest advise the community in the project development phase.<br><br>**VVBs:**<br>**a)** Ensure that all communication materials presented as evidence of engagement are in the **community's native language.**
Communities face socio-economic challenges that affect their rights of self-determination, such as structural violence in the collective territories, external pressures that incentivize extractive activities, and gender inequality.	*Communities, project developers, and standards have advanced a series of actions to overcome socio-economic challenges and impediments to self-determination, including a focus on economic activities that support a just transition towards conservation models, gender equity, and education.*	
**a) Communities can face pressure from illegal groups** when information on developing a project in the community is widespread.<br><br>**b)** To ensure the **legitimacy of any assembly or consultative body**, considerable obstacles must be overcome, including the physical distance between population centers, the presence of illegal organizations, and the existence of informal political borders in the project area. These issues are exacerbated in indigenous communities.<br><br>**c) Lack of economic opportunities** has forced IPALC to engage in activities, legal and illegal, that harm conservation goals.<br><br>**d)** In some Indigenous communities, **women have limited participation in the project's formulation** unless gender-based programs are implemented.	**a)** Some projects have **supported the transition from deforestation and illegal activities to sustainable development models**. Projects report cases in which members dedicated to illegal logging or mining have transitioned to forest ranger activities, green businesses, ecotourism, or mangrove nurseries, among other things.<br><br>**b)** Some projects include a **line of work in control and surveillance of deforestation** and offer training to community members in forest ranger activities to control the territory.<br><br>**c) Projects offer educational opportunities** to youth by improving educational facilities, providing graduate scholarships, and hiring teachers. These provide alternatives to youth and keep them away from criminal activities.<br><br>**d)** A developer created **outreach and educational materials in the**	**Government:**<br>**a) Create financial mechanisms** for IPALC to support the economic transition towards sustainable development models that reduce deforestation and prioritize forest conservation in collective territories.<br><br>**b) Advance in the implementation of the Escazu agreement** to materialize actions related to access to justice and the protection of IPALC as vulnerable environmental leaders.<br><br>**IPALC:**<br>**a) Adopt lines of work within the projects' investment plans to support the economic transition** towards sustainable development models, including resources for developing green businesses, ecotourism activities, nurseries and forest rangers, and forest monitoring. Invest in strengthening the local technical capacities and infrastructure to manage the businesses.

**e) Developers have had different experiences interacting with Indigenous and Afro-descendant communities** and reported finding it more challenging to align the project's goals with Indigenous communities because of language barriers, broader worldview differences, and governance practices.<br><br>**f)** To meet their immediate needs, **communities sometimes request additional resources from developers** outside the project's original scope. If these requests are not fulfilled, they can hinder the project's progress.	**communities' native languages,** including videos, games, and forms of communication that support oral traditions.<br><br>**e)** Standards are developing **tools to measure projects' social impact. One registry has developed a tool for developers to show SDG compliance with defined criteria and indicators.** For example, some standards require gender equity in project development.<br><br>**f)** Communities are **strengthening the role and leadership of women in Indigenous communities** by including them in decision-making processes, supporting the development of women-owned businesses and women-led forestry and botanical activities, and recovering and maintaining ancestral knowledge traditionally held by women.	**b) Develop yearly work plans** to create employment opportunities for community members engaged in sectors not aligned with conservation and promote a transition towards green businesses, monitoring, and conservation activities. This plan should be approved by the assembly and aligned with the life plan.<br><br>**Developers:**<br>**a)** Produce communication materials in the **community's native language** and incorporate **communication practices based on oral traditions.**<br><br>**VVBs:**<br>**a)** Ensure that all communication materials presented as evidence of engagement are in the **native language of the community.**<br><br>**Registries and Standards:**<br>**a) Require projects to show alignment with the SDGs**, particularly gender equity, strengthening governance, and ending poverty.<br><br>**b) Adopt tools to measure the social impact of projects**, including compliance with SDGs with defined criteria and indicators and requesting supporting evidence during the verification process.
Communities with weak governance mechanisms experience institutional instability, lack of agreement and control in using collective resources, limited trust and legitimacy, and limited resources to develop life plans and internal governance norms to address the challenges of carbon projects.	*Communities, international cooperation agencies, and developers are working to strengthen governance capacities through the investment in governance mechanisms, including the development of life plans, conducting assemblies with the presence of the public ministry, creating accountability committees, and working through trust funds with collectively agreed investment lines to manage resources.*	

<table>
<tr>
<td rowspan="2">**a) Frequent shifts in leadership positions** within Indigenous communities can create obstacles to developing REDD+ projects due to priority changes within the project formulation timeframe.

b) IPALC have limited capacities and resources to formulate life plans and internal norms to promote transformational change. When carbon forestry projects and resources enter the territory, life plans and internal laws are usually adjusted to accommodate the new financial and implementation capacities and to determine which economic activities are allowed.

c) There are some cases in which ethnic authorities convene assemblies to decide on participation in a carbon market project **without providing the necessary information in advance for community members** to make informed decisions. Sometimes, the communities are informed about the REDD+ project in the same instance where they are expected to vote on it.

d) The absence of a mechanism guaranteeing that decisions about carbon projects are collectively agreed upon creates instability and the possibility for opposing actors to block a project's development or enter a slow mediation process with the Ministry of Interior.

e) Some ethnic authorities have the faculties to decide on participation in carbon projects **without using mechanisms that guarantee broad community participation in the decisions**. This creates conditions where leaders have been reported to receive resources to influence their project decisions.

f) The Internal governance mechanisms of some</td>
<td rowspan="2">**a)** International cooperation and some developers have incorporated the **strengthening of self-governance capacities as a fundamental activity in developing projects** beyond the technical requirements of REDD+ projects.

b) Communities often **invest project resources to sustain a more robust governance structure**, increasing the capacity to manage the territory more autonomously.

c) Projects structured through international cooperation have longer periods of education and engagement with IPALC, helping them **align the investments with their life plans**, including, in some cases, reviewing and updating life plans.

d) Communities th**at give ethnic authority less autonomy and have a robust system of internal consultative bodies** for decision-making face less instability in developing carbon projects.

e) Community assemblies as mechanisms for decision-making have been effective mechanisms to guarantee collective agreement in carbon projects, mainly when there is a presence of the public ministry (personería, procuraduría, defensoría del pueblo) acting as observer and proper minutes and assistance records. Frequent assemblies help to guarantee legitimacy.

f) The sector association, Asocarbono, implemented an **evaluation and ethics committee** that conducts relevant investigations if a community presents a complaint regarding a project.

g) Developers sometimes use the</td>
<td>**Government:**
a) Strengthen the Ministry of Interior's role and capacities in providing technical assistance to IPALC to update life plans and internal governance norms, focusing on strengthening collective agreements for the use of resources and accountability mechanisms.</td>
</tr>
<tr>
<td>**IPALC:**
a) Establish an association of IPALC and ethnic organizations for carbon projects to share best practices among communities associated with the alignment of investment and life plans; promoting partnerships for the development of high-quality and more attractive projects for the international market; promoting partners with the best track records working with communities; collecting and sharing information about sales and benefit-sharing to reduce information asymmetries; and facilitating access to financial mechanisms to overcome entry barriers, among others.

b) Position experienced leaders to continue to work closely with the community council governing boards, particularly during leadership transitions.

c) Prioritize engagement in carbon projects that lead towards full ownership and the development of local capacities to increase community participation in project activities and benefit-sharing over the lifetime of a project with the overall purpose of achieving a higher degree of autonomy and self-governance. Communities should rely less on private partners for general aspects of the project development and contract external partners for specific aspects of projects where specialized expertise is required.</td>
</tr>
</table>

**communities are not appropriate for participating in the design, approval, and implementation of REDD+ projects** due to their lack of agility and difficulties guaranteeing the participation of the majority of the community in extensive territories.	role of a **community liaison to enhance their capacity to transmit information and build long-term relationships in the territories**. This person is most effective when selected by the community.<br><br>**h) Accountability committees led by ethnic authorities** to track the investment of resources from carbon forestry projects facilitate the community's understanding and involvement in carbon projects, reinforcing internal governance and financial education.<br><br>**i) Trust funds** with specific lines of investment and mandatory proof of expenditure before releasing the funds are commonly used by developers as a mechanism to guarantee transparency and alignment in the use of resources with previously agreed priorities.	**d) Communities with smaller land extensions should partner with other IPALC** to make their projects more attractive **to** international buyers.<br><br>**e) Include penalties in the internal norms** for ethnic authorities who mismanage or divert resources from collectively agreed priorities.<br><br>**f) Adopt the practice of community assemblies with the presence of the public ministry** to reach collective agreement on the milestones of a carbon project, including the decision to start a project, benefit-sharing, and lines of investment, among others.<br><br>**g) Establish accountability assemblies and community oversight groups** where ethnic authorities periodically report to the community on the use and management of economic resources generated by the projects.
		**Developers:**<br>**a) Adopt the practice of creating trust funds** with specific investment lines and mandatory proof of expenditure before releasing the funds to guarantee transparency and alignment in the use of resources with previously agreed-upon priorities.<br><br>**b) Adopt the practice of community assemblies with the presence of the public ministry** to reach a collective agreement on the milestones of a carbon project, including the decision to start a project, benefit-sharing, and lines of investment, among others.
		**Registries and Standards:**<br>**a) Require community governance goals** to be incorporated into projects, and establish indicators to measure progress.
